# Supplementary material for: Metabolic capabilities of key rumen microbiota drive methane emissions in cattle
Source: mSystems. 2025 Sep 22;10(10):e00601-25. doi: 10.1128/msystems.00601-25 (PMC12542661; doi:10.1128/msystems.00601-25)
Supplement: Supplemental material — Supplemental figures and tables. [file msystems.00601-25-s0001.pdf]

Supplementary Materials

Title

Metabolic Capabilities of Key Rumen Microbiota Drive Methane Emissions in Cattle

Authors:

Wanxin Lai[1], Antton Alberdi[2], Andy Leu[3], Arturo V. P. de Leon[4], Carl M. Kobel [4], Velma T. E. Aho[4], Rainer Roehe[5], Phil B. Pope[1,3,4], Torgeir R. Hvidsten[1]

1 Faculty of Chemistry, Biotechnology and Food Science, Norwegian University of Life Sciences, Ås, Norway
2 Center for Evolutionary Hologenomics, Globe Institute, University of Copenhagen, Copenhagen, Denmark
3 Centre for Microbiome Research, Faculty of Health, School of Biomedical Sciences, Queensland University of Technology, Translational Research Institute, Woolloongabba, Australia
4 Faculty of Biosciences, Norwegian University of Life Sciences, Ås, Norway
5 Department of Agriculture, Horticulture and Engineering Sciences, Scotland's Rural College, Edinburgh, UK

Content

Fig. S1 HMSC modelled variables (Timepoint, Breed, Emission, Pro. 3
Fig. S2 Variance partition of variables modelled by HMSC. 4
Fig. S3 KEGG pathway analysis of propionate metabolism...................................... 5
Fig. S4 The KEGG pathway of amino acids degradation. More doma.. 6
Fig. S5 The KEGG pathway of glycolysis, and the Methylmalonyl-Co 7

Table S1 - Variance explained by our model 8
Table S2 - T-test showing LEC 8
Table S3 - HEC-MAGs predicted by HMSC 9
Table S4 - LEC-MAGs predicted by HMSC 16
Table S5 - Distribution of Samples Across Animal Breeds 21

References 22

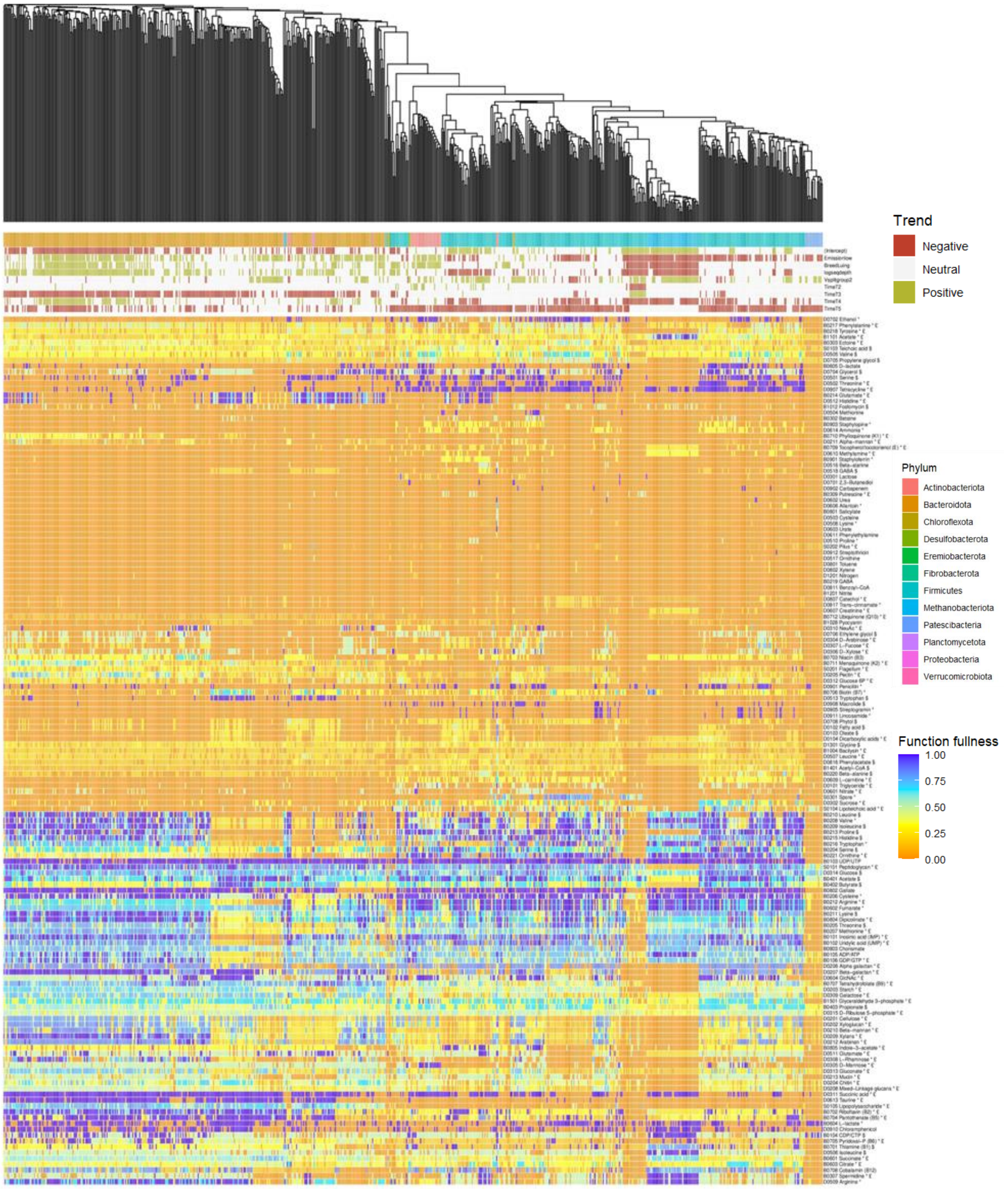

Fig. S1 HMSC modelled variables (Timepoint, Breed, Emission, Protozoa-driven clustering/RCT-types (Vsplitgroup2) and covariate (logseqdepth)) and strain level functional landscape shows a highly complex functional profile. Function fullness refers to the extent to which a gene's functional domains are present and complete, indicating the potential to perform its associated biological functions. Asterisk “*” on the right mark emission-focussed functional traits with BH-adjusted p < 0.05, “£” and “$” represents traits passed the multiple testing when “Breed” and “Protozoa-driven clustering (Vsplitgroup2)”/"RCT-type" were used

as the focal variables instead. The colour scale of functional fullness represents the completeness of gene domains for each functional trait of every MAG in the rumen gut. More details about the protozoa-driven clustering can be found in (Kobel et al., 2024). Additional supplementary documentations for model convergence such as potential scale reduction factor (psrf) and effective sample size (ESS) were included in the supplementary materials.

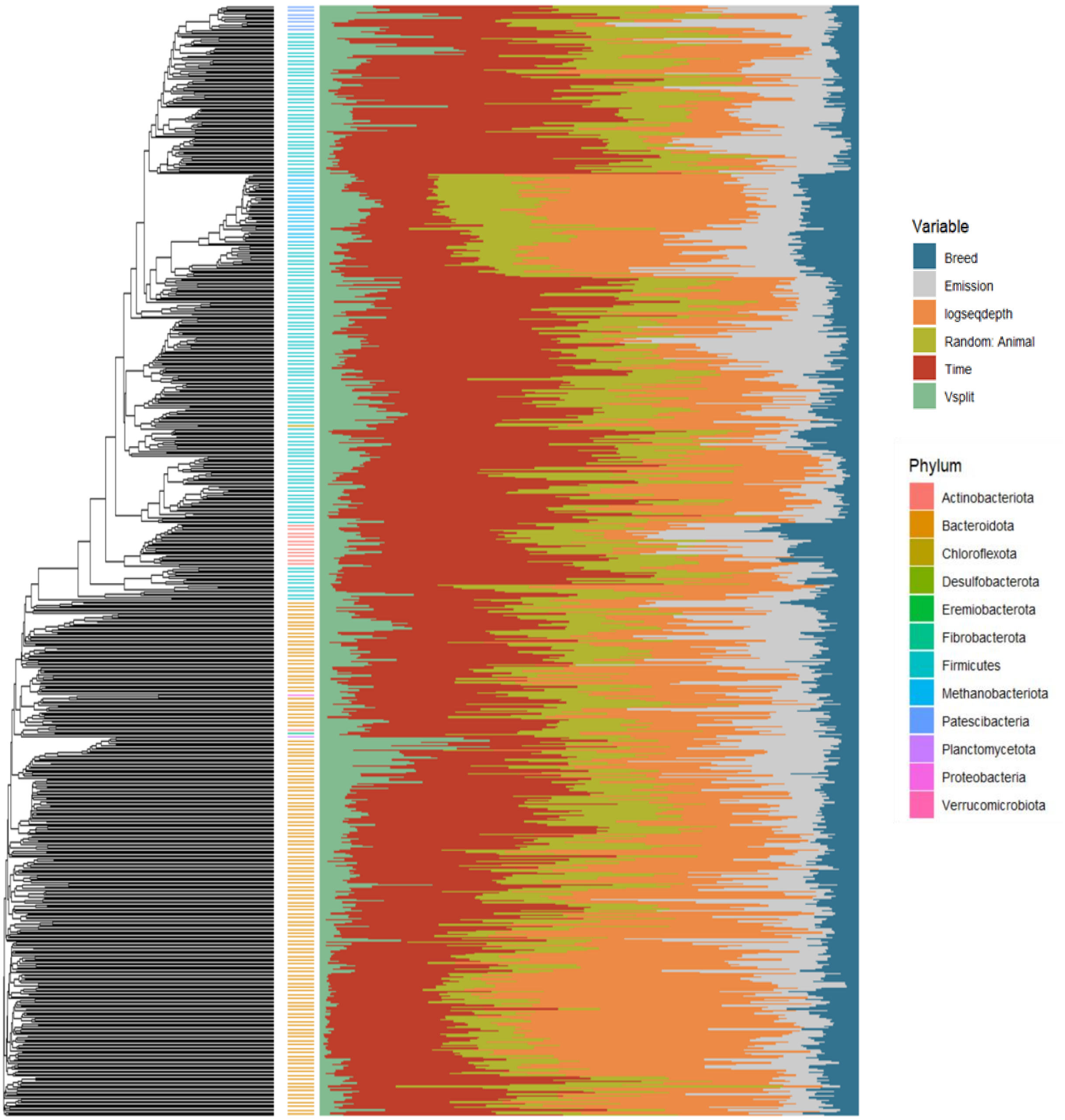

Fig. S2 Variance partition of variables modelled by HMSC.

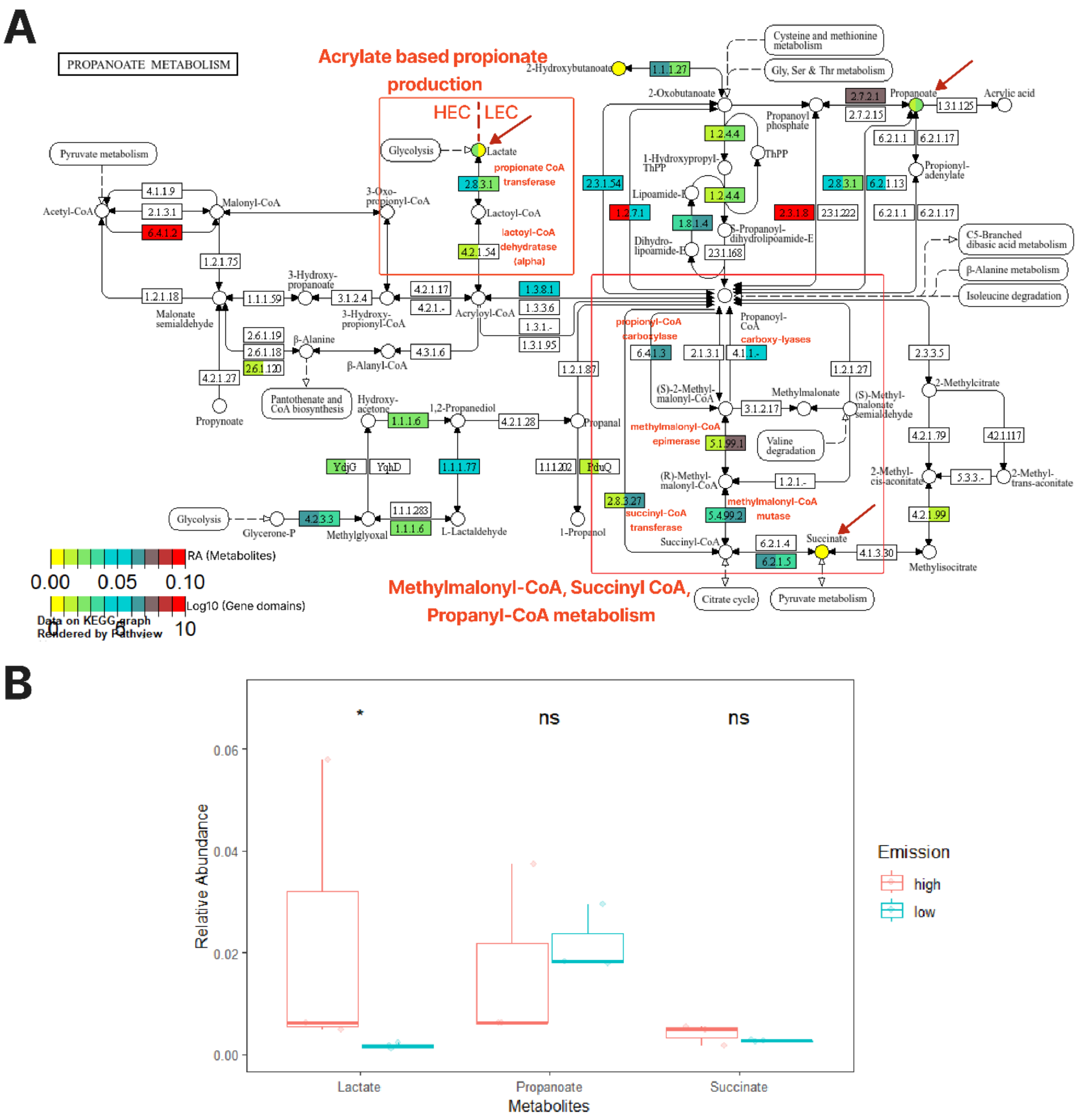

Fig S3: **A** KEGG pathway analysis of propionate metabolism, comparing enzyme domain abundance in MAGs associated with HEC (left) and LEC (right). The upper red square highlights lactate metabolism/acrylate-based propionate production in both groups. Elevated L-lactate accumulation and enzyme domains in HEC suggest more active lactate producers compared to LEC. Succinate-based propionate metabolism and their key enzymes such as Methylmalonyl-CoA transferase and epimerase were higher in LEC than HEC (lower red square). Boxes are coloured by the number of gene domains (log10-scale) found in the MAGs positively associated with HEC (left) and LEC (right). Circles represent the mean normalised relative abundance of metabolites (ranging from 0 to 0.1). **B** Boxplot illustrating the relative abundance of three metabolites highlighted in the propanoate metabolism pathway. Metabolites from Timepoint 5 (T5) were selected for pathway visualisation due to

their temporal proximity to CH4 measurements. Among these, lactate showed statistical significance (p-value < 0.05).

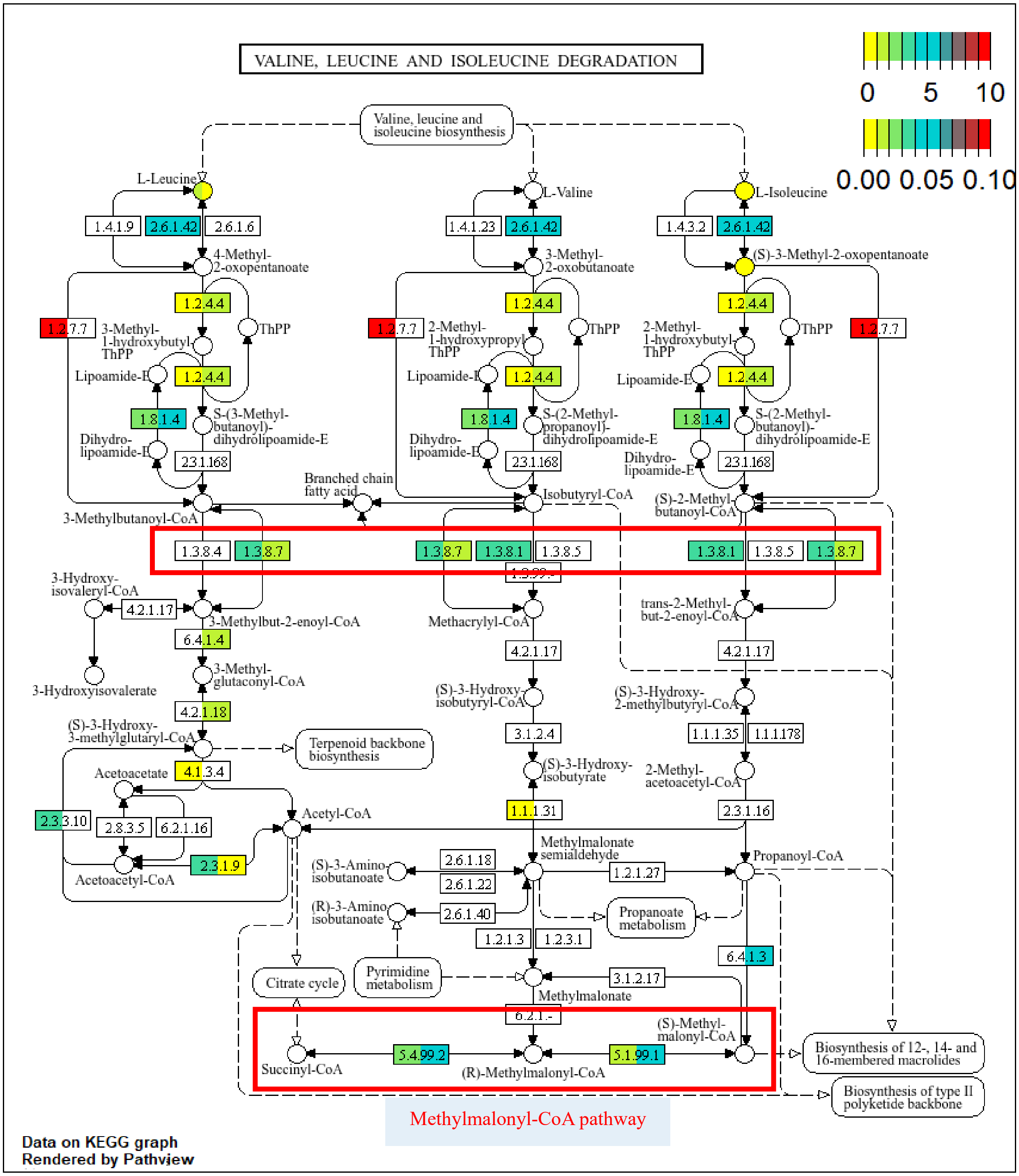

Fig. S4 The KEGG pathway of amino acids degradation. More domains for key enzyme in the acrylate-based propionate pathway (EC 1.3.8.7 in the top red box) were found in HEC-MAGs than LEC-MAGs. Methylmalonyl-CoA pathway, where LEC-MAGs possessed more gene domains than HEC-MAGs, are highlighted by the lower red square. The boxes are coloured

by the number of gene domains (log10-scale) found in the MAGs with a positive response to the HEC (left side) and LEC (right side), and the average relative abundance of metabolites (circle).

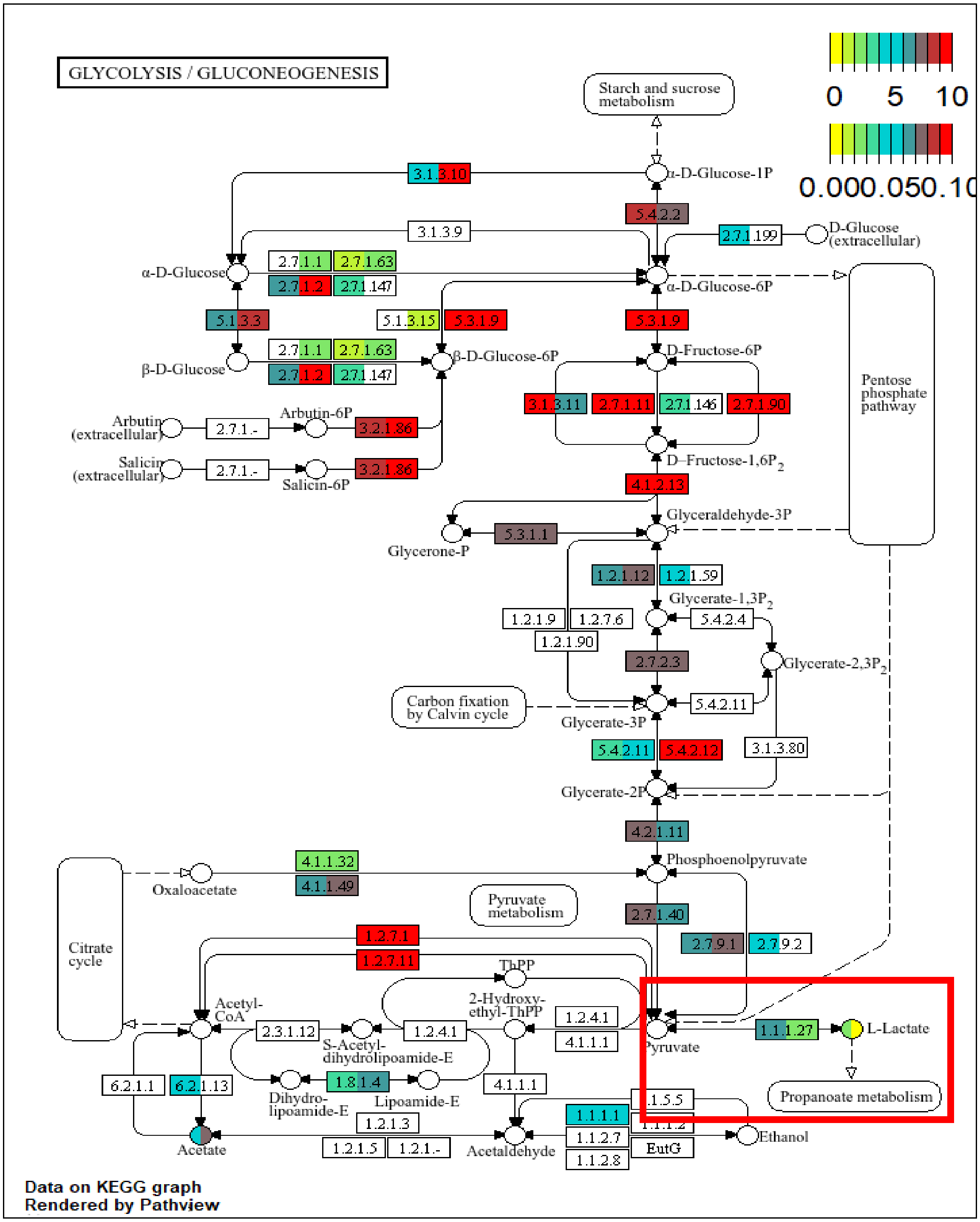

Fig. S5 The KEGG pathway of glycolysis, and the L-lactate dehydrogenase (EC 1.1.1.27) where HEC-MAGs possessed more gene domains than LEC-MAGs. The boxes are coloured by the number of gene domains (log10-scale) found in the MAGs with a positive response

to the HEC (left side) and LEC (right side). More lactate metabolites were found in HEC than LEC.

Table S1 Variance explained by our model is distributed across multiple factors: breed contributes a mean of 6.82% (SD = 2.94), while emission accounts for 13.59%. Log sequencing depth (covariates) explains a mean of 24.23% (SD = 13.79). The random effect of the animal itself contributes 15.43% (SD = 6.46). Time accounts for the largest proportion, with a mean contribution of 33.24% (SD = 10.98). Finally, rumen community type (RCT)/Vsplit explains 6.69% of the variance (SD = 4.73).

**Variable**	**Mean**	**sd**
Breed	6.8181	2.9426
Emission	13.5912	8.2741
Logseqdepth	24.2292	13.7817
Random: Animal	15.4282	6.4563
Time	33.2399	10.9832
Vsplit	6.6932	4.7278

Table S2 T-test showing LEC has significantly different average metabolic capacity index (MCI) compared to HEC (p-value: 2.056e-09). The mean value of LEC (0.2206823) is higher than HEC (0.1834293).

Welch Two Sample t-test		
t		-6.1678
df		324.9
95 % Confidence Interval	Lower	-0.0491
	Upper	-0.0253
Mean (HEC, LEC)		0.1834, 0.2207
p		2.056e-09

Table S3 Highly prevalent 191 HEC-MAGs predicted by HMSC and corresponding MCI.

	genome	variable	value	trend	Domain	Phylum	Genus	Species	Completeness	Contamination	Genome_Size	mag_size	MCI
1	Control_HighE.FMIC.metabat.1087	Emission low	0.03	Negative	Prokaryota	Methanobacteriota	Methanobrevibacter	sp	99.68	1.95	2158473	2.16	0.186563
2	Control_HighE.FMIC.metabat.1122	Emission low	0.018333	Negative	Prokaryota	Firmicutes	Ruminococcus	sp	98.92	0.19	2642713	2.64	0.245521
3	Control_HighE.FMIC.metabat.548	Emission low	0.003333	Negative	Prokaryota	Firmicutes	RUG12438	sp	87.88	9.77	1776445	1.78	0.057813
4	Control_HighE.FMIC.metabat.82	Emission low	0.01	Negative	Prokaryota	Methanobacteriota	Methanosphaera	sp	83.88	4.96	1826173	1.83	0.161146
5	Control_HighE.FMIC.vae_11763	Emission low	0.013333	Negative	Prokaryota	Methanobacteriota	Methanobrevibacter	sp	79.33	0.56	1900032	1.9	0.167292
6	Control_HighE.FMIC.vae_15920	Emission low	0.021667	Negative	Prokaryota	Methanobacteriota	Methanobrevibacter	sp	95.63	0.35	2575129	2.58	0.185156
7	Control_HighE.FMIC.vae_17425	Emission low	0.015	Negative	Prokaryota	Firmicutes	RUG754	sp	93.41	2.39	1759354	1.76	0.206771
8	Control_HighE.FMIC.vae_17855	Emission low	0.025	Negative	Prokaryota	Firmicutes	RUG11890	sp	87.96	7.79	1759646	1.76	0.055365
9	Control_HighE.FMIC.vae_19524	Emission low	0.05	Negative	Prokaryota	Firmicutes	RGIG5755	sp	94.82	0.5	1634139	1.63	0.194271
10	Control_HighE.FMIC.vae_20654	Emission low	0.005	Negative	Prokaryota	Firmicutes	RUG343	sp	87.4	5.47	1089104	1.09	0.059115
11	Control_HighE.FMIC.vae_39473	Emission low	0	Negative	Prokaryota	Firmicutes	UBA3789	sp	76.56	5	1054869	1.05	0.032656
12	Control_HighE.FMIC.vae_4410	Emission low	0.003333	Negative	Prokaryota	Firmicutes	Ruminococcus	sp	74.79	1.58	1929667	1.93	0.204375
13	Control_HighE.FMIC.vae_4598	Emission low	0.01	Negative	Prokaryota	Methanobacteriota	Methanobrevibacter	sp	96.54	0.65	2230229	2.23	0.188438
14	Control_HighE.FMIC.vae_7000	Emission low	0.05	Negative	Prokaryota	Methanobacteriota	Methanobrevibacter	sp	95.4	0.52	2412364	2.41	0.178906
15	Control_HighE.metabat.1003	Emission low	0.095	Negative	Prokaryota	Firmicutes	Bulleidia	sp	94.71	5.3	2501998	2.5	0.230156
16	Control_HighE.metabat.212	Emission low	0	Negative	Prokaryota	Firmicutes	RUG11224	sp	72.04	7.39	1467628	1.47	0.05276
17	Control_HighE.metabat.244	Emission low	0.098333	Negative	Prokaryota	Firmicutes	UBA4285	sp	88.47	4.15	1756746	1.76	0.187917
18	Control_HighE.metabat.418	Emission low	0.013333	Negative	Prokaryota	Firmicutes	CAG-791	sp	77.55	1.58	2246982	2.25	0.205208
19	Control_HighE.metabat.535	Emission low	0.095	Negative	Prokaryota	Firmicutes	CAG-791	sp	93.11	4.61	2982030	2.98	0.234323
20	Control_HighE.metabat.648	Emission low	0	Negative	Prokaryota	Firmicutes	RUG12438	sp	74.98	7.87	1319751	1.32	0.035
21	Control_HighE.metabat.714	Emission low	0.008333	Negative	Prokaryota	Firmicutes	RGIG5612	sp	69.83	1	2400746	2.4	0.211927
22	Control_HighE.metabat.908	Emission low	0.036667	Negative	Prokaryota	Firmicutes	Ruminococcus	sp	76.15	1.68	1964837	1.96	0.17776
23	Control_HighE.metabat.951	Emission low	0	Negative	Prokaryota	Firmicutes	RGIG5622	sp	78.18	5.49	1205069	1.21	0.074323

24	Control_HighE.metabat.981	Emission low	0.053333	Negative	Prokaryota	Firmicutes	UBA4285	sp	77.12	8.66	1443527	1.44	0.151302
25	Control_HighE.vamb.10491	Emission low	0.041667	Negative	Prokaryota	Patescibacteria	UBA2834	sp	72.4	4.81	908999	0.91	0.03224
26	Control_HighE.vamb.1419	Emission low	0.005	Negative	Prokaryota	Firmicutes	Porcincola	sp	97.46	1.45	3080350	3.08	0.313229
27	Control_HighE.vamb.22097	Emission low	0.066667	Negative	Prokaryota	Firmicutes	RUG842	sp	98.88	0.47	1887067	1.89	0.228073
28	Control_HighE.vamb.24290	Emission low	0.038333	Negative	Prokaryota	Methanobacteriota	Methanobrevibacter	sp	99.11	0.39	1835362	1.84	0.18401
29	Control_HighE.vamb.3520	Emission low	0.006667	Negative	Prokaryota	Firmicutes	CAG-465	sp	82.73	5.46	1547336	1.55	0.073385
30	Control_HighE.vamb.4799	Emission low	0.025	Negative	Prokaryota	Firmicutes	RUG842	sp	78.92	1.05	1556610	1.56	0.198021
31	Control_HighE.vamb.5322	Emission low	0.038333	Negative	Prokaryota	Methanobacteriota	Methanobrevibacter	sp	86.24	4.17	2345852	2.35	0.176406
32	Control_HighE.vamb.6994	Emission low	0.001667	Negative	Prokaryota	Firmicutes	CAG-791	sp	76.69	8.2	2533491	2.53	0.259948
33	Control_HighE.vamb.9230	Emission low	0.015	Negative	Prokaryota	Firmicutes	Ruminococcus	sp	90.69	1.23	1708374	1.71	0.20901
34	Control_LowE.FMIC.vae_13449	Emission low	0.048333	Negative	Prokaryota	Methanobacteriota	Methanobrevibacter	sp	79.73	2.6	1819661	1.82	0.15974
35	Control_LowE.FMIC.vae_2667	Emission low	0.095	Negative	Prokaryota	Firmicutes	Porcincola	sp	78.75	4.44	2627685	2.63	0.231719
36	Control_LowE.FMIC.vae_3283	Emission low	0.028333	Negative	Prokaryota	Methanobacteriota	Methanobrevibacter	sp	83.86	2.14	1675096	1.68	0.168594
37	Control_LowE.FMIC.vae_6917	Emission low	0.088333	Negative	Prokaryota	Firmicutes	UBA1066	sp	93.55	3.29	3347848	3.35	0.27349
38	Control_LowE.FMIC.vae_7488	Emission low	0.056667	Negative	Prokaryota	Firmicutes	RGIG5755	sp	83.49	7.41	1731263	1.73	0.192135
39	Control_LowE.FMIC.vae_9096	Emission low	0.008333	Negative	Prokaryota	Firmicutes	CAG-791	sp	79.7	2.48	1917966	1.92	0.185365
40	Control_LowE.metabat.424	Emission low	0.013333	Negative	Prokaryota	Methanobacteriota	Methanobrevibacter	sp	81.61	2.46	2319692	2.32	0.16724
41	Control_LowE.vamb.1303	Emission low	0.013333	Negative	Prokaryota	Methanobacteriota	Methanobrevibacter	sp	84.6	0.52	2057959	2.06	0.173333
42	Control_LowE.vamb.2792	Emission low	0	Negative	Prokaryota	Firmicutes	UBA5578	sp	80.88	8.57	2321835	2.32	0.048438
43	Control_MidE.FMIC.metabat.114	Emission low	0.041667	Negative	Prokaryota	Methanobacteriota	Methanobrevibacter	thaueri	94.16	0.15	1805046	1.81	0.179219
44	Control_MidE.FMIC.metabat.528	Emission low	0.035	Negative	Prokaryota	Methanobacteriota	Methanobrevibacter	sp	90.3	3.42	2218377	2.22	0.179635
45	Control_MidE.FMIC.metabat.550	Emission low	0	Negative	Prokaryota	Firmicutes	Ruminococcus	sp	78.11	0.44	1596896	1.6	0.18849
46	Control_MidE.FMIC.vae_2406	Emission low	0.038333	Negative	Prokaryota	Firmicutes	Ruminococcus	sp	97.8	2.17	2241851	2.24	0.230313
47	Control_MidE.FMIC.vae_3227	Emission low	0.001667	Negative	Prokaryota	Firmicutes	Ruminococcus	sp	89.23	4.99	2037206	2.04	0.21724
48	Control_MidE.FMIC.vae_3316	Emission low	0.036667	Negative	Prokaryota	Methanobacteriota	Methanobrevibacter	sp	79.51	8.51	2310802	2.31	0.144219
49	Control_MidE.FMIC.vae_6141	Emission low	0	Negative	Prokaryota	Firmicutes	UBA3789	sp	77.51	8.02	1644891	1.64	0.027969
50	Control_MidE.metabat.116	Emission low	0	Negative	Prokaryota	Firmicutes	RUG343	sp	74.87	10.94	1144376	1.14	0.039427
51	Control_MidE.metabat.156	Emission low	0.055	Negative	Prokaryota	Patescibacteria	UBA2834	sp	72.47	3.83	949665	0.95	0.031823

52	Control_MidE.metabat.185	Emission low	0.01	Negative	Prokaryota	Patescibacteria	Nanosyncoccus	sp	73.72	8.26	845803	0.85	0.043542
53	Control_MidE.metabat.305	Emission low	0.053333	Negative	Prokaryota	Methanobacteriota	Methanobrevibacter	sp	77.11	7.12	1923374	1.92	0.155781
54	Control_MidE.metabat.306	Emission low	0.088333	Negative	Prokaryota	Firmicutes	UBA3766	sp	78.39	8.71	2803120	2.8	0.226094
55	Control_MidE.metabat.34	Emission low	0.04	Negative	Prokaryota	Patescibacteria	UBA2834	sp	91.21	0.38	685755	0.69	0.030104
56	Control_MidE.metabat.342	Emission low	0.018333	Negative	Prokaryota	Methanobacteriota	Methanobrevibacter	sp	71.87	9.18	2126148	2.13	0.146927
57	Control_MidE.metabat.459	Emission low	0.048333	Negative	Prokaryota	Firmicutes	Hornefia	sp	73.95	4.72	1851465	1.85	0.145885
58	Control_MidE.metabat.470	Emission low	0.02	Negative	Prokaryota	Patescibacteria	UBA2834	sp	71.1	2.38	829721	0.83	0.03901
59	Control_MidE.metabat.57	Emission low	0.026667	Negative	Prokaryota	Firmicutes	Firm-16	sp	90.88	9.36	2674937	2.67	0.224375
60	Control_MidE.metabat.693	Emission low	0.021667	Negative	Prokaryota	Firmicutes	CAG-791	sp	97.51	1.44	3543486	3.54	0.293281
61	Control_MidE.metabat.713	Emission low	0.005	Negative	Prokaryota	Firmicutes	Ruminococcus	sp	100	1.24	2365398	2.37	0.247292
62	Control_MidE.metabat.791	Emission low	0.065	Negative	Prokaryota	Firmicutes	CAG-791	sp	96.18	3.17	3018965	3.02	0.28151
63	Control_MidE.metabat.863	Emission low	0.048333	Negative	Prokaryota	Firmicutes	Porcincola	sp	74.21	2.68	2068644	2.07	0.228646
64	Control_MidE.metabat.98	Emission low	0.005	Negative	Prokaryota	Firmicutes	Ruminococcus	sp	99.71	2.31	1809668	1.81	0.219323
65	Control_MidE.vamb.1519	Emission low	0.016667	Negative	Prokaryota	Methanobacteriota	Methanobrevibacter	sp	86.34	0.8	2275117	2.28	0.175885
66	Control_MidE.vamb.19838	Emission low	0.071667	Negative	Prokaryota	Firmicutes	RGIG5755	sp	95.95	0.05	2366084	2.37	0.240781
67	Control_MidE.vamb.2682	Emission low	0.026667	Negative	Prokaryota	Patescibacteria	UBA2834	sp	99.15	0.35	1069092	1.07	0.038333
68	Control_MidE.vamb.42943	Emission low	0.038333	Negative	Prokaryota	Firmicutes	RUG12519	sp	100	1.81	1228720	1.23	0.157292
69	Control_MidE.vamb.5528	Emission low	0.063333	Negative	Prokaryota	Firmicutes	Porcincola	sp	93.07	0.62	3417766	3.42	0.298438
70	Control_MidE.vamb.5745	Emission low	0.003333	Negative	Prokaryota	Firmicutes	CAG-791	sp	78.24	1.64	1467006	1.47	0.209375
71	Control_MidE.vamb.5826	Emission low	0.033333	Negative	Prokaryota	Firmicutes	CAG-791	sp	87.1	4.42	2669939	2.67	0.21875
72	Control_MidE.vamb.683	Emission low	0.07	Negative	Prokaryota	Firmicutes	Eubacterium	sp	98.88	3.33	2288543	2.29	0.239167
73	HighE_A02_bin431	Emission low	0.008333	Negative	Prokaryota	Firmicutes	UBA1066	sp	80.33	8.5	2683669	2.68	0.235469
74	HighE_A16_bin199	Emission low	0.01	Negative	Prokaryota	Firmicutes	UBA1066	sp	95.52	0.83	2484182	2.48	0.258385
75	HighE_A16_bin225	Emission low	0.048333	Negative	Prokaryota	Methanobacteriota	Methanobrevibacter	sp	94.74	2.99	4275406	4.28	0.170677
76	HighE_A16_bin487	Emission low	0.015	Negative	Prokaryota	Firmicutes	UBA1066	sp	81.68	3.5	2826643	2.83	0.251875
77	HighE_A16_bin69	Emission low	0.013333	Negative	Prokaryota	Methanobacteriota	Methanobrevibacter	sp	100	0.61	2708344	2.71	0.193021
78	HighE_A29_bin575	Emission low	0.005	Negative	Prokaryota	Firmicutes	UBA1066	sp	77.66	1.76	2672215	2.67	0.229948
79	HighE_A42_bin274	Emission low	0	Negative	Prokaryota	Firmicutes	UBA3789	sp	73.37	8.99	1195100	1.2	0.037448

80	HighE_A42_bin470	Emission low	0	Negative	Prokaryota	Firmicutes	CAG-791	sp	77.53	5.04	2162495	2.16	0.228438
81	HighE_A42_bin499	Emission low	0.078333	Negative	Prokaryota	Firmicutes	UBA3633	sp	94.85	3.82	2042009	2.04	0.135313
82	HighE_A51_bin184	Emission low	0.006667	Negative	Prokaryota	Firmicutes	UBA1066	sp	70.99	3.8	2072134	2.07	0.171354
83	HighE_A51_bin506	Emission low	0	Negative	Prokaryota	Firmicutes	CAG-791	sp	78.09	1.61	1377931	1.38	0.218646
84	HighE_A60_bin212	Emission low	0.038333	Negative	Prokaryota	Firmicutes	RUG12519	sp	92.53	0.29	1116744	1.12	0.108333
85	HighE_A60_bin320	Emission low	0.035	Negative	Prokaryota	Methanobacteriota	Methanobrevibacter	sp	84.46	0.43	1961512	1.96	0.166094
86	HighE_A63_bin198	Emission low	0.008333	Negative	Prokaryota	Firmicutes	UBA1066	sp	81.64	5.89	2566231	2.57	0.233854
87	HighE_A63_bin86	Emission low	0.006667	Negative	Prokaryota	Firmicutes	Ruminococcus	sp	94.56	6.89	2478076	2.48	0.201302
88	HighE_A67_bin223	Emission low	0.001667	Negative	Prokaryota	Firmicutes	CAG-791	sp	71.87	2.8	1703167	1.7	0.24375
89	LowE_A06_bin126	Emission low	0.008333	Negative	Prokaryota	Firmicutes	UBA1066	sp	76.35	6.91	2662699	2.66	0.224583
90	LowE_A06_bin75	Emission low	0.065	Negative	Prokaryota	Methanobacteriota	Methanobrevibacter	sp	96.58	0.27	2187161	2.19	0.195052
91	LowE_A06_bin76	Emission low	0	Negative	Prokaryota	Firmicutes	CAG-791	sp	77.7	8.83	2200615	2.2	0.247813
92	LowE_A08_bin165	Emission low	0.02	Negative	Prokaryota	Firmicutes	UBA1066	sp	90.76	8.22	3201269	3.2	0.298698
93	LowE_A15_bin101	Emission low	0.005	Negative	Prokaryota	Patescibacteria	UBA2834	sp	98.08	0.15	963715	0.96	0.041406
94	LowE_A15_bin132	Emission low	0	Negative	Prokaryota	Patescibacteria	Nanosyncoccus	sp	88.34	2.6	915683	0.92	0.059844
95	LowE_A15_bin198	Emission low	0.003333	Negative	Prokaryota	Firmicutes	RUG13615	sp	75.52	9.28	1910087	1.91	0.16724
96	LowE_A15_bin199	Emission low	0.015	Negative	Prokaryota	Firmicutes	UBA1066	sp	93.01	0.15	2059167	2.06	0.277188
97	LowE_A15_bin243	Emission low	0	Negative	Prokaryota	Firmicutes	Ruminococcus	sp	87.11	2.54	1719456	1.72	0.173281
98	LowE_A15_bin271	Emission low	0.006667	Negative	Prokaryota	Firmicutes	UBA1066	sp	88.82	6.45	2508102	2.51	0.247604
99	LowE_A15_bin558	Emission low	0.026667	Negative	Prokaryota	Firmicutes	CAG-603	sp	99.67	0.75	2374702	2.37	0.242708
100	LowE_A15_bin564	Emission low	0	Negative	Prokaryota	Firmicutes	Ruminococcus	sp	72.3	4.51	1842898	1.84	0.206198
101	LowE_A15_bin609	Emission low	0	Negative	Prokaryota	Firmicutes	CAG-269	sp	75.86	3.38	1240973	1.24	0.050052
102	LowE_A15_bin90	Emission low	0.005	Negative	Prokaryota	Firmicutes	CAG-603	sp	86.15	3.61	2863674	2.86	0.214583
103	LowE_A25_bin388	Emission low	0.018333	Negative	Prokaryota	Patescibacteria	UBA2834	sp	98.13	0.21	978209	0.98	0.046719
104	LowE_A28_bin294	Emission low	0.023333	Negative	Prokaryota	Firmicutes	CAG-791	sp	94.88	3.01	2582012	2.58	0.262188
105	LowE_A35_bin78	Emission low	0.021667	Negative	Prokaryota	Methanobacteriota	Methanobrevibacter	sp	77.56	0.73	1523094	1.52	0.150365
106	LowE_A45_bin286	Emission low	0.041667	Negative	Prokaryota	Firmicutes	UBA1066	sp	82.75	1.56	2601266	2.6	0.219427
107	LowE_A45_bin318	Emission low	0.015	Negative	Prokaryota	Firmicutes	RUG099	sp	76.88	7.73	1599241	1.6	0.112448

108	LowE_A45_bin324	Emission low	0.028333	Negative	Prokaryota	Firmicutes	UBA1066	sp	77.7	1.87	2447118	2.45	0.204271
109	LowE_A48_bin382	Emission low	0	Negative	Prokaryota	Firmicutes	CAG-791	sp	79.95	9.41	2340910	2.34	0.228281
110	LowE_A55_bin47	Emission low	0	Negative	Prokaryota	Firmicutes	Ruminococcus	sp	99.79	3.61	2224554	2.22	0.212552
111	LowE_A61_bin168	Emission low	0	Negative	Prokaryota	Patescibacteria	Nanosyncoccus	sp	95.12	0.29	811136	0.81	0.048594
112	LowE_A61_bin174	Emission low	0.02	Negative	Prokaryota	Firmicutes	Kandleria	vitulina	100	0.99	2109030	2.11	0.261198
113	LowE_A75_bin210	Emission low	0.001667	Negative	Prokaryota	Firmicutes	UBA1066	sp	78.76	5.71	2344127	2.34	0.235938
114	Treatment_HighE.FMIC.metabat.368	Emission low	0.008333	Negative	Prokaryota	Methanobacteriota	Methanosphaera	sp	99.36	0.34	2288383	2.29	0.178177
115	Treatment_HighE.FMIC.metabat.537	Emission low	0.091667	Negative	Prokaryota	Firmicutes	RGIG5755	sp	80.78	1.91	1731587	1.73	0.192188
116	Treatment_HighE.FMIC.metabat.701	Emission low	0.055	Negative	Prokaryota	Firmicutes	Succiniclasticum	ruminis	78.63	6.65	2233418	2.23	0.223073
117	Treatment_HighE.FMIC.vae_1257	Emission low	0.01	Negative	Prokaryota	Methanobacteriota	Methanobrevibacter	sp	99.98	0.3	2228176	2.23	0.18625
118	Treatment_HighE.FMIC.vae_12660	Emission low	0.013333	Negative	Prokaryota	Methanobacteriota	Methanobrevibacter	sp	93.37	0.62	2424470	2.42	0.181667
119	Treatment_HighE.FMIC.vae_13557	Emission low	0	Negative	Prokaryota	Firmicutes	Porcincola	sp	78.28	2.37	2471345	2.47	0.260625
120	Treatment_HighE.FMIC.vae_19859	Emission low	0.036667	Negative	Prokaryota	Firmicutes	UBA1066	sp	96.36	0.32	2604945	2.6	0.258385
121	Treatment_HighE.FMIC.vae_2724	Emission low	0.003333	Negative	Prokaryota	Methanobacteriota	Methanobrevibacter	sp	91.31	1.29	2118139	2.12	0.178073
122	Treatment_HighE.FMIC.vae_5124	Emission low	0.045	Negative	Prokaryota	Methanobacteriota	Methanobrevibacter	sp	97.24	1.15	2497507	2.5	0.176198
123	Treatment_HighE.FMIC.vae_6550	Emission low	0.005	Negative	Prokaryota	Firmicutes	Ruminococcus	sp	84.19	6.47	1894326	1.89	0.191094
124	Treatment_HighE.FMIC.vae_6867	Emission low	0.003333	Negative	Prokaryota	Firmicutes	CAG-791	sp	91.65	1.45	3919708	3.92	0.247813
125	Treatment_HighE.FMIC.vae_7306	Emission low	0.003333	Negative	Prokaryota	Firmicutes	UBA3789	sp	89.63	0.11	1365492	1.37	0.054323
126	Treatment_HighE.FMIC.vae_7328	Emission low	0	Negative	Prokaryota	Firmicutes	Ruminococcus	sp	91.58	2.75	1915267	1.92	0.217969
127	Treatment_HighE.MaxBin.0052	Emission low	0	Negative	Prokaryota	Firmicutes	CAG-791	sp	74.47	11.5	1300056	1.3	0.129271
128	Treatment_HighE.MaxBin.0055	Emission low	0.006667	Negative	Prokaryota	Firmicutes	Streptococcus	sp	70.64	4.86	1281953	1.28	0.149115
129	Treatment_HighE.MaxBin.0080	Emission low	0.001667	Negative	Prokaryota	Firmicutes	RUG343	sp	70.67	3.58	712122	0.71	0.035885
130	Treatment_HighE.metabat.141	Emission low	0.06	Negative	Prokaryota	Firmicutes	Ruminococcus	sp	78.25	2.03	2139896	2.14	0.203542
131	Treatment_HighE.metabat.704	Emission low	0.025	Negative	Prokaryota	Methanobacteriota	Methanobrevibacter	sp	81.87	0.09	1624898	1.62	0.159688
132	Treatment_HighE.metabat.705	Emission low	0.028333	Negative	Prokaryota	Methanobacteriota	Methanobrevibacter	sp	95.39	1.57	2612913	2.61	0.192969
133	Treatment_HighE.metabat.73	Emission low	0.043333	Negative	Prokaryota	Firmicutes	Firm-16	sp	71.41	7.1	2228412	2.23	0.219531
134	Treatment_HighE.metabat.769	Emission low	0.011667	Negative	Prokaryota	Firmicutes	RGIG5612	sp	74.04	7.58	2576497	2.58	0.211667
135	Treatment_HighE.vamb.10918	Emission low	0.048333	Negative	Prokaryota	Firmicutes	UBA3738	sp	93.36	1.8	1801348	1.8	0.21651

136	Treatment_HighE.vamb.1450	Emission low	0.061667	Negative	Prokaryota	Firmicutes	Ruminococcus	sp	99.7	1.18	2037899	2.04	0.24224
137	Treatment_HighE.vamb.2674	Emission low	0.011667	Negative	Prokaryota	Firmicutes	RUG12519	sp	78.9	4.48	1369747	1.37	0.073802
138	Treatment_HighE.vamb.3007	Emission low	0.008333	Negative	Prokaryota	Firmicutes	RUG099	sp	88.4	0.62	2065397	2.07	0.244375
139	Treatment_HighE.vamb.3590	Emission low	0.015	Negative	Prokaryota	Firmicutes	Ruminococcus	sp	97.34	6.34	2400525	2.4	0.231979
140	Treatment_HighE.vamb.4533	Emission low	0.081667	Negative	Prokaryota	Firmicutes	UBA1066	sp	70.35	6.35	2553919	2.55	0.19651
141	Treatment_HighE.vamb.4928	Emission low	0.025	Negative	Prokaryota	Methanobacteriota	Methanobrevibacter	sp	78.41	2.33	1783867	1.78	0.154531
142	Treatment_HighE.vamb.5405	Emission low	0	Negative	Prokaryota	Firmicutes	RGIG5612	sp	74.81	0.55	1977842	1.98	0.196406
143	Treatment_HighE.vamb.5571	Emission low	0.015	Negative	Prokaryota	Methanobacteriota	Methanosphaera	sp	85.78	1.22	2129992	2.13	0.155208
144	Treatment_HighE.vamb.6535	Emission low	0	Negative	Prokaryota	Patescibacteria	Nanosyncoccus	sp	78.33	10.04	945597	0.95	0.037344
145	Treatment_LowE.FMIC.metabat.1014	Emission low	0	Negative	Prokaryota	Firmicutes	Ruminococcus	sp	85.24	0.96	2130806	2.13	0.228438
146	Treatment_LowE.FMIC.metabat.314	Emission low	0.078333	Negative	Prokaryota	Firmicutes	Hornefia	sp	87.69	1.16	2069601	2.07	0.221771
147	Treatment_LowE.FMIC.metabat.407	Emission low	0.051667	Negative	Prokaryota	Firmicutes	Ruminococcus	sp	79.83	0.78	1863579	1.86	0.211458
148	Treatment_LowE.FMIC.metabat.559	Emission low	0.005	Negative	Prokaryota	Firmicutes	Porcincola	sp	92.72	4.32	2562828	2.56	0.284271
149	Treatment_LowE.FMIC.metabat.614	Emission low	0.005	Negative	Prokaryota	Firmicutes	CAG-791	sp	70.72	2.67	2049556	2.05	0.25224
150	Treatment_LowE.FMIC.metabat.638	Emission low	0.013333	Negative	Prokaryota	Firmicutes	RUG13615	sp	72.72	5.12	1747321	1.75	0.202708
151	Treatment_LowE.FMIC.metabat.642	Emission low	0	Negative	Prokaryota	Firmicutes	Ruminococcus	sp	100	3.71	2158102	2.16	0.23401
152	Treatment_LowE.FMIC.metabat.969	Emission low	0.005	Negative	Prokaryota	Firmicutes	CAG-603	sp	99.48	0.01	2418335	2.42	0.242969
153	Treatment_LowE.FMIC.vae_15581	Emission low	0.011667	Negative	Prokaryota	Firmicutes	CAG-791	sp	86.7	1.19	2276756	2.28	0.257344
154	Treatment_LowE.FMIC.vae_24646	Emission low	0.013333	Negative	Prokaryota	Firmicutes	Ruminococcus	sp	87.6	1.97	2262783	2.26	0.198958
155	Treatment_LowE.FMIC.vae_3707	Emission low	0.041667	Negative	Prokaryota	Firmicutes	UBA636	sp	93.45	0.84	2132950	2.13	0.17099
156	Treatment_LowE.FMIC.vae_3878	Emission low	0.038333	Negative	Prokaryota	Firmicutes	Ruminococcus	sp	89.49	1.66	1911679	1.91	0.194323
157	Treatment_LowE.FMIC.vae_4876	Emission low	0.08	Negative	Prokaryota	Actinobacteriota	Bifidobacterium	ruminantium	80.22	0.66	1726124	1.73	0.228385
158	Treatment_LowE.FMIC.vae_6376	Emission low	0.051667	Negative	Prokaryota	Firmicutes	UBA3766	sp	90.68	5.96	2482943	2.48	0.253281
159	Treatment_LowE.FMIC.vae_6961	Emission low	0.056667	Negative	Prokaryota	Firmicutes	RUG099	sp	70.84	5.14	1662589	1.66	0.181354
160	Treatment_LowE.FMIC.vae_7783	Emission low	0.018333	Negative	Prokaryota	Firmicutes	RUG099	sp	79.45	3.65	1955351	1.96	0.235938
161	Treatment_LowE.FMIC.vae_9229	Emission low	0.058333	Negative	Prokaryota	Firmicutes	RUG099	sp	74.21	1.04	1584699	1.58	0.188906
162	Treatment_LowE.metabat.205	Emission low	0.076667	Negative	Prokaryota	Methanobacteriota	Methanobrevibacter	sp	85.57	0.66	2177398	2.18	0.177604
163	Treatment_LowE.metabat.369	Emission low	0.025	Negative	Prokaryota	Actinobacteriota	Bifidobacterium	globosum	99.32	0.08	1898806	1.9	0.268594

164	Treatment_LowE.metabat.39	Emission low	0.001667	Negative	Prokaryota	Firmicutes	Ruminococcus	sp	74.57	3.62	1762911	1.76	0.145833
165	Treatment_LowE.metabat.461	Emission low	0	Negative	Prokaryota	Firmicutes	CAG-791	sp	84.44	5.83	2175247	2.18	0.255573
166	Treatment_LowE.metabat.550	Emission low	0.011667	Negative	Prokaryota	Methanobacteriota	Methanobrevibacter	sp	72.84	3.89	2029821	2.03	0.158802
167	Treatment_LowE.metabat.589	Emission low	0.015	Negative	Prokaryota	Firmicutes	Ruminococcus	sp	73.26	2.4	1850308	1.85	0.178125
168	Treatment_LowE.metabat.641	Emission low	0.063333	Negative	Prokaryota	Firmicutes	CAG-791	sp	83.65	2.5	2003084	2	0.265573
169	Treatment_LowE.metabat.655	Emission low	0.018333	Negative	Prokaryota	Firmicutes	Sharpea	azabuensis	99.93	1.64	2527643	2.53	0.287083
170	Treatment_LowE.metabat.707	Emission low	0.051667	Negative	Prokaryota	Firmicutes	RUG099	sp	81.91	1.45	1727552	1.73	0.21474
171	Treatment_LowE.metabat.81	Emission low	0.018333	Negative	Prokaryota	Patescibacteria	UBA2834	sp	97.63	2.14	964193	0.96	0.039479
172	Treatment_LowE.metabat.838	Emission low	0	Negative	Prokaryota	Firmicutes	CAG-791	sp	94.54	2.81	2483290	2.48	0.285365
173	Treatment_LowE.vamb.1459	Emission low	0.006667	Negative	Prokaryota	Firmicutes	Ruminococcus	sp	100	1.52	2406887	2.41	0.227396
174	Treatment_LowE.vamb.4309	Emission low	0.008333	Negative	Prokaryota	Methanobacteriota	Methanobrevibacter	sp	81.56	6.05	1953264	1.95	0.165469
175	Treatment_LowE.vamb.6266	Emission low	0.023333	Negative	Prokaryota	Methanobacteriota	Methanobrevibacter	sp	94	3.8	3493776	3.49	0.17849
176	Treatment_LowE.vamb.7146	Emission low	0.05	Negative	Prokaryota	Methanobacteriota	Methanobrevibacter	sp	74.71	6.07	2002946	2	0.152969
177	Treatment_LowE.vamb.8605	Emission low	0.05	Negative	Prokaryota	Firmicutes	Porcincola	sp	79.17	2.51	2268605	2.27	0.215365
178	Treatment_MidE.FMIC.metabat.192	Emission low	0.023333	Negative	Prokaryota	Methanobacteriota	Methanobrevibacter	sp	78.6	7.03	2089790	2.09	0.1575
179	Treatment_MidE.FMIC.metabat.375	Emission low	0.058333	Negative	Prokaryota	Methanobacteriota	Methanobrevibacter	sp	94.62	0.78	2251450	2.25	0.180729
180	Treatment_MidE.FMIC.vae_1303	Emission low	0.003333	Negative	Prokaryota	Bacteroidota	Prevotella	sp	99.94	0.13	3088522	3.09	0.305365
181	Treatment_MidE.FMIC.vae_3248	Emission low	0.043333	Negative	Prokaryota	Methanobacteriota	Methanobrevibacter	sp	77.6	1.2	2327372	2.33	0.168229
182	Treatment_MidE.FMIC.vae_9438	Emission low	0	Negative	Prokaryota	Firmicutes	UBA3789	sp	97.3	5.96	1733599	1.73	0.055938
183	Treatment_MidE.metabat.218	Emission low	0	Negative	Prokaryota	Firmicutes	UBA3789	sp	66.54	10.01	1763504	1.76	0.066042
184	Treatment_MidE.metabat.480	Emission low	0	Negative	Prokaryota	Firmicutes	CAG-791	sp	98.08	2.26	2768459	2.77	0.283698
185	Treatment_MidE.metabat.531	Emission low	0	Negative	Prokaryota	Firmicutes	Ruminococcus	sp	81.66	1.3	1788358	1.79	0.233021
186	Treatment_MidE.metabat.559	Emission low	0.008333	Negative	Prokaryota	Methanobacteriota	Methanobrevibacter	millerae	69.86	1.82	2034941	2.03	0.133281
187	Treatment_MidE.metabat.584	Emission low	0.006667	Negative	Prokaryota	Firmicutes	CAG-791	sp	94.23	2.5	3107527	3.11	0.280052
188	Treatment_MidE.metabat.602	Emission low	0.021667	Negative	Prokaryota	Methanobacteriota	Methanobrevibacter	sp	85.2	7.92	2338825	2.34	0.176406
189	Treatment_MidE.vamb.2269	Emission low	0.023333	Negative	Prokaryota	Methanobacteriota	Methanobrevibacter	sp	76.86	5.82	2028615	2.03	0.143542
190	Treatment_MidE.vamb.2688	Emission low	0.02	Negative	Prokaryota	Firmicutes	Ruminococcus	sp	96.82	7.64	2210913	2.21	0.201615
191	Treatment_MidE.vamb.3845	Emission low	0.081667	Negative	Prokaryota	Firmicutes	Porcincola	sp	99.49	1.99	2818033	2.82	0.280677

Table S4 Highly prevalent 220 LEC-MAGs predicted by HMSC and corresponding MCI

	genome	X	variable	value	trend	Domain	Phylum	Genus	Species	Completeness	Contamination	Genome_Size	mag_size	MCI
1	Control_HighE.FMIC.metabat.1124	110	Emissionlow	0.98	Positive	Prokaryota	Actinobacteriota	SIG37	sp	85.86	0.95	1853390	1.85	0.176875
2	Control_HighE.FMIC.metabat.163	134	Emissionlow	0.968333	Positive	Prokaryota	Bacteroidota	Prevotella	sp	70.2	4.24	2687664	2.69	0.253385
3	Control_HighE.FMIC.metabat.175	179	Emissionlow	0.938333	Positive	Prokaryota	Actinobacteriota	UBA9715	sp	90.04	2.17	2521466	2.52	0.216875
4	Control_HighE.FMIC.metabat.725	25	Emissionlow	0.996667	Positive	Prokaryota	Bacteroidota	Cryptobacteroides	sp	94.36	1.73	2098744	2.1	0.22375
5	Control_HighE.FMIC.metabat.78	39	Emissionlow	0.995	Positive	Prokaryota	Bacteroidota	RF16	sp	80.7	9.09	2543393	2.54	0.148542
6	Control_HighE.FMIC.vae_10344	4	Emissionlow	1	Positive	Prokaryota	Bacteroidota	Bact-11	sp	94.92	1.57	2188172	2.19	0.178385
7	Control_HighE.FMIC.vae_10953	77	Emissionlow	0.986667	Positive	Prokaryota	Bacteroidota	Prevotella	sp	85.48	2.63	2686584	2.69	0.254115
8	Control_HighE.FMIC.vae_12301	164	Emissionlow	0.95	Positive	Prokaryota	Bacteroidota	RF16	sp	80.8	2.55	1837405	1.84	0.154375
9	Control_HighE.FMIC.vae_12874	181	Emissionlow	0.936667	Positive	Prokaryota	Bacteroidota	Sodaliphilus	sp	91.71	1.38	3503982	3.5	0.214688
10	Control_HighE.FMIC.vae_217	204	Emissionlow	0.916667	Positive	Prokaryota	Bacteroidota	RF16	sp	87.17	1.02	2134312	2.13	0.170208
11	Control_HighE.FMIC.vae_2310	15	Emissionlow	0.998333	Positive	Prokaryota	Bacteroidota	RF16	sp	85.69	0.44	2380818	2.38	0.204323
12	Control_HighE.FMIC.vae_2898	190	Emissionlow	0.926667	Positive	Prokaryota	Bacteroidota	RF16	sp	83.48	3.9	2316291	2.32	0.157656
13	Control_HighE.FMIC.vae_31029	197	Emissionlow	0.923333	Positive	Prokaryota	Actinobacteriota	UBA9715	sp	89.97	0.9	2440369	2.44	0.210938
14	Control_HighE.FMIC.vae_3380	33	Emissionlow	0.995	Positive	Prokaryota	Bacteroidota	Prevotella	sp	72.33	7.17	2395960	2.4	0.204479
15	Control_HighE.FMIC.vae_3703	62	Emissionlow	0.99	Positive	Prokaryota	Bacteroidota	Prevotella	sp	76.28	3.35	3226954	3.23	0.245833
16	Control_HighE.FMIC.vae_3740	160	Emissionlow	0.951667	Positive	Prokaryota	Bacteroidota	Sodaliphilus	sp	82.79	1.15	2818655	2.82	0.190521
17	Control_HighE.FMIC.vae_3815	192	Emissionlow	0.925	Positive	Prokaryota	Bacteroidota	Bact-11	sp	88.54	2.38	2576527	2.58	0.204115
18	Control_HighE.FMIC.vae_4090	71	Emissionlow	0.988333	Positive	Prokaryota	Bacteroidota	Prevotella	sp	70.13	4.32	2697427	2.7	0.242448
19	Control_HighE.FMIC.vae_41843	157	Emissionlow	0.953333	Positive	Prokaryota	Bacteroidota	UBA4372	sp	98.18	1.29	2579396	2.58	0.256042
20	Control_HighE.FMIC.vae_420	7	Emissionlow	1	Positive	Prokaryota	Bacteroidota	UBA4372	sp	90.56	2.01	2315646	2.32	0.236979
21	Control_HighE.FMIC.vae_4482	89	Emissionlow	0.985	Positive	Prokaryota	Actinobacteriota	UBA1367	sp	83.37	3.56	2721686	2.72	0.235625
22	Control_HighE.FMIC.vae_4750	150	Emissionlow	0.96	Positive	Prokaryota	Bacteroidota	Prevotella	sp	79.8	3.53	3262300	3.26	0.247708
23	Control_HighE.FMIC.vae_5247	166	Emissionlow	0.948333	Positive	Prokaryota	Firmicutes	QANA01	sp	79	0.27	1406677	1.41	0.185104
24	Control_HighE.FMIC.vae_5374	23	Emissionlow	0.996667	Positive	Prokaryota	Bacteroidota	Prevotella	sp	84.81	4.41	3786953	3.79	0.273229
25	Control_HighE.FMIC.vae_5428	202	Emissionlow	0.92	Positive	Prokaryota	Bacteroidota	Prevotella	sp	73.93	7.1	3265755	3.27	0.22
26	Control_HighE.FMIC.vae_5540	1	Emissionlow	1	Positive	Prokaryota	Bacteroidota	Prevotella	sp	78.06	3.41	3034811	3.03	0.229271
27	Control_HighE.FMIC.vae_7012	79	Emissionlow	0.986667	Positive	Prokaryota	Bacteroidota	Prevotella	sp	85.5	2.19	2725855	2.73	0.255156
28	Control_HighE.FMIC.vae_7071	82	Emissionlow	0.986667	Positive	Prokaryota	Bacteroidota	Prevotella	sp	83.94	3.37	2704628	2.7	0.271198
29	Control_HighE.FMIC.vae_7403	38	Emissionlow	0.995	Positive	Prokaryota	Bacteroidota	Limimorpha	sp	71.35	4.62	2663394	2.66	0.151094
30	Control_HighE.FMIC.vae_7409	36	Emissionlow	0.995	Positive	Prokaryota	Bacteroidota	Prevotella	sp	73.19	5.38	2549884	2.55	0.226719
31	Control_HighE.FMIC.vae_7922	127	Emissionlow	0.971667	Positive	Prokaryota	Bacteroidota	Bact-11	sp	84.72	1.1	2157490	2.16	0.183438
32	Control_HighE.FMIC.vae_8067	53	Emissionlow	0.991667	Positive	Prokaryota	Actinobacteriota	RUG440	sp	83.62	3.26	4375899	4.38	0.232083
33	Control_HighE.metabat.1053	184	Emissionlow	0.933333	Positive	Prokaryota	Bacteroidota	UBA1756	sp	72.58	4.36	2092541	2.09	0.133646
34	Control_HighE.metabat.218	142	Emissionlow	0.966667	Positive	Prokaryota	Bacteroidota	Cryptobacteroides	sp	70.65	2.07	2052018	2.05	0.148698
35	Control_HighE.metabat.25	175	Emissionlow	0.941667	Positive	Prokaryota	Bacteroidota	UBA4372	sp	70.25	2.16	3239094	3.24	0.242135
36	Control_HighE.metabat.307	193	Emissionlow	0.925	Positive	Prokaryota	Bacteroidota	Limimorpha	sp	73.54	6.39	2936746	2.94	0.178281
37	Control_HighE.metabat.500	158	Emissionlow	0.953333	Positive	Prokaryota	Bacteroidota	UBA4372	sp	72.29	5.05	2572807	2.57	0.20901
38	Control_HighE.metabat.505	91	Emissionlow	0.985	Positive	Prokaryota	Bacteroidota	Cryptobacteroides	sp	73.77	6.49	2371108	2.37	0.173333
39	Control_HighE.metabat.596	121	Emissionlow	0.975	Positive	Prokaryota	Actinobacteriota	RUG440	sp	72.86	4.54	2653358	2.65	0.186042

40	Control_HighE.metabat.641	170	Emissionlow	0.943333	Positive	Prokaryota	Bacteroidota	Prevotella	sp	74.03	7.22	2779731	2.78	0.230104
41	Control_HighE.metabat.692	161	Emissionlow	0.951667	Positive	Prokaryota	Bacteroidota	Enterocola	sp	86.07	4.55	2026990	2.03	0.182552
42	Control_HighE.metabat.733	135	Emissionlow	0.968333	Positive	Prokaryota	Bacteroidota	Prevotella	ruminicola	80.23	4.46	2863616	2.86	0.267188
43	Control_HighE.metabat.764	140	Emissionlow	0.966667	Positive	Prokaryota	Firmicutes	Limivicinus	sp	85.74	2.78	2345324	2.35	0.209427
44	Control_HighE.metabat.770	11	Emissionlow	0.998333	Positive	Prokaryota	Actinobacteriota	UBA1367	sp	80.35	2.57	3356387	3.36	0.218594
45	Control_HighE.metabat.776	151	Emissionlow	0.96	Positive	Prokaryota	Bacteroidota	Egerieousia	sp	75.05	7.39	1621538	1.62	0.125208
46	Control_HighE.metabat.953	125	Emissionlow	0.973333	Positive	Prokaryota	Bacteroidota	Cryptobacteroides	sp	94.9	1.54	2877627	2.88	0.235052
47	Control_HighE.vamb.1502	191	Emissionlow	0.925	Positive	Prokaryota	Bacteroidota	Prevotella	sp	79.14	4.09	2327719	2.33	0.212708
48	Control_HighE.vamb.2058	201	Emissionlow	0.921667	Positive	Prokaryota	Firmicutes	GCA-900199385	sp	91.88	2.22	2856387	2.86	0.275677
49	Control_HighE.vamb.4016	55	Emissionlow	0.991667	Positive	Prokaryota	Bacteroidota	Bact-11	sp	74.62	2.57	1843802	1.84	0.162031
50	Control_HighE.vamb.5136	211	Emissionlow	0.91	Positive	Prokaryota	Bacteroidota	Cryptobacteroides	sp	75.64	1.96	2022184	2.02	0.149896
51	Control_HighE.vamb.5162	163	Emissionlow	0.95	Positive	Prokaryota	Firmicutes	GCA-900199385	sp	78.78	2.65	2980798	2.98	0.25026
52	Control_HighE.vamb.5341	169	Emissionlow	0.945	Positive	Prokaryota	Firmicutes	GCA-900199385	sp	72.71	0.31	2208106	2.21	0.219792
53	Control_HighE.vamb.62	212	Emissionlow	0.908333	Positive	Prokaryota	Bacteroidota	Cryptobacteroides	sp	76.47	3.91	2208227	2.21	0.171927
54	Control_HighE.vamb.6531	168	Emissionlow	0.946667	Positive	Prokaryota	Bacteroidota	Cryptobacteroides	sp	70.28	2.43	2005701	2.01	0.134844
55	Control_HighE.vamb.6640	24	Emissionlow	0.996667	Positive	Prokaryota	Bacteroidota	Cryptobacteroides	sp	86.66	0.68	2231428	2.23	0.147292
56	Control_HighE.vamb.6667	14	Emissionlow	0.998333	Positive	Prokaryota	Bacteroidota	F23-D06	sp	86.84	4.36	2802311	2.8	0.194531
57	Control_HighE.vamb.6932	5	Emissionlow	1	Positive	Prokaryota	Bacteroidota	RF16	sp	79.98	0.68	1836302	1.84	0.123125
58	Control_HighE.vamb.7932	59	Emissionlow	0.991667	Positive	Prokaryota	Bacteroidota	RF16	sp	75.24	3.56	1812477	1.81	0.138125
59	Control_HighE.vamb.8032	155	Emissionlow	0.955	Positive	Prokaryota	Bacteroidota	Cryptobacteroides	sp	84.65	3.3	2583471	2.58	0.196094
60	Control_HighE.vamb.8901	122	Emissionlow	0.975	Positive	Prokaryota	Bacteroidota	UBA1217	sp	95.03	1.11	2404612	2.4	0.184323
61	Control_HighE.vamb.911	146	Emissionlow	0.963333	Positive	Prokaryota	Proteobacteria	UBA2804	sp	86.95	3.21	2744769	2.74	0.251875
62	Control_LowE.FMIC.metabat.177	94	Emissionlow	0.985	Positive	Prokaryota	Bacteroidota	UBA2918	sp	85.58	5.28	2337673	2.34	0.224531
63	Control_LowE.FMIC.vae_3089	118	Emissionlow	0.976667	Positive	Prokaryota	Bacteroidota	Prevotella	sp	83.14	7.9	2902413	2.9	0.282292
64	Control_LowE.FMIC.vae_3090	75	Emissionlow	0.988333	Positive	Prokaryota	Bacteroidota	UBA1711	sp	95.06	3.14	3121460	3.12	0.197969
65	Control_LowE.vamb.1369	111	Emissionlow	0.98	Positive	Prokaryota	Bacteroidota	UBA1786	sp	87.41	3.15	3561939	3.56	0.285469
66	Control_LowE.vamb.1803	154	Emissionlow	0.956667	Positive	Prokaryota	Bacteroidota	RUG11690	sp	84.9	3.44	2601725	2.6	0.193021
67	Control_LowE.vamb.3501	203	Emissionlow	0.918333	Positive	Prokaryota	Bacteroidota	Cryptobacteroides	sp	79.77	1.7	2280661	2.28	0.199688
68	Control_MidE.FMIC.metabat.742	2	Emissionlow	1	Positive	Prokaryota	Bacteroidota	Cryptobacteroides	sp	97.29	0.4	2318699	2.32	0.21026
69	Control_MidE.FMIC.vae_1013	6	Emissionlow	1	Positive	Prokaryota	Bacteroidota	RF16	sp	80.47	2.55	1926466	1.93	0.149844
70	Control_MidE.FMIC.vae_1699	205	Emissionlow	0.915	Positive	Prokaryota	Bacteroidota	Prevotella	sp	85.24	2.94	3497683	3.5	0.27526
71	Control_MidE.FMIC.vae_2312	76	Emissionlow	0.988333	Positive	Prokaryota	Bacteroidota	RF16	sp	94.56	4.45	2636848	2.64	0.164323
72	Control_MidE.FMIC.vae_2515	98	Emissionlow	0.983333	Positive	Prokaryota	Bacteroidota	Sodaliphilus	sp	92.88	4.61	3468680	3.47	0.209896
73	Control_MidE.FMIC.vae_2580	100	Emissionlow	0.983333	Positive	Prokaryota	Bacteroidota	RF16	sp	77.55	2.26	2699945	2.7	0.159896
74	Control_MidE.FMIC.vae_29659	97	Emissionlow	0.983333	Positive	Prokaryota	Bacteroidota	Prevotella	sp	99.76	1.36	3947301	3.95	0.304479
75	Control_MidE.FMIC.vae_3351	84	Emissionlow	0.986667	Positive	Prokaryota	Bacteroidota	Prevotella	sp	82.48	5.82	2935145	2.94	0.264948
76	Control_MidE.FMIC.vae_376	27	Emissionlow	0.996667	Positive	Prokaryota	Bacteroidota	UBA1217	sp	85.98	0.18	2093252	2.09	0.174271
77	Control_MidE.FMIC.vae_501	51	Emissionlow	0.991667	Positive	Prokaryota	Bacteroidota	Prevotella	sp	76.78	3.48	3417767	3.42	0.247188
78	Control_MidE.FMIC.vae_6049	123	Emissionlow	0.973333	Positive	Prokaryota	Actinobacteriota	Parafannyhessea	sp	92.55	0.29	2372076	2.37	0.216146
79	Control_MidE.FMIC.vae_693	17	Emissionlow	0.996667	Positive	Prokaryota	Bacteroidota	Prevotella	sp	77.92	9.13	2757523	2.76	0.228229
80	Control_MidE.FMIC.vae_7096	42	Emissionlow	0.993333	Positive	Prokaryota	Actinobacteriota	UBA1367	sp	97.14	4.31	2795004	2.8	0.261042

81	Control_MidE.FMIC.vae_7191	30	Emissionlow	0.996667	Positive	Prokaryota	Bacteroidota	UBA4334	sp	78.67	4.7	2957951	2.96	0.274479
82	Control_MidE.FMIC.vae_829	165	Emissionlow	0.95	Positive	Prokaryota	Bacteroidota	RF16	sp	76.38	2.59	1822760	1.82	0.133854
83	Control_MidE.metabat.177	13	Emissionlow	0.998333	Positive	Prokaryota	Bacteroidota	Cryptobacteroides	sp	70.59	7.09	2336963	2.34	0.158281
84	Control_MidE.metabat.207	186	Emissionlow	0.933333	Positive	Prokaryota	Bacteroidota	UBA3839	sp	80.56	8.08	3599947	3.6	0.234635
85	Control_MidE.metabat.325	101	Emissionlow	0.981667	Positive	Prokaryota	Bacteroidota	Prevotella	sp	72.35	1.95	3554119	3.55	0.265469
86	Control_MidE.metabat.364	18	Emissionlow	0.996667	Positive	Prokaryota	Bacteroidota	Prevotella	sp	99.62	4.08	3498045	3.5	0.306979
87	Control_MidE.metabat.438	141	Emissionlow	0.966667	Positive	Prokaryota	Bacteroidota	Cryptobacteroides	sp	71.74	7.64	2009413	2.01	0.18276
88	Control_MidE.metabat.510	34	Emissionlow	0.995	Positive	Prokaryota	Bacteroidota	Prevotella	sp	88.78	1.71	3381844	3.38	0.292031
89	Control_MidE.metabat.518	213	Emissionlow	0.908333	Positive	Prokaryota	Bacteroidota	RF16	sp	81.79	7.73	2246079	2.25	0.143646
90	Control_MidE.metabat.568	113	Emissionlow	0.978333	Positive	Prokaryota	Bacteroidota	Prevotella	sp	71.44	3.06	2355112	2.36	0.21724
91	Control_MidE.metabat.571	31	Emissionlow	0.995	Positive	Prokaryota	Bacteroidota	Prevotella	sp	92.47	0.53	2538127	2.54	0.275885
92	Control_MidE.metabat.788	48	Emissionlow	0.991667	Positive	Prokaryota	Bacteroidota	Prevotella	sp	76.57	9.3	2728800	2.73	0.203333
93	Control_MidE.metabat.796	147	Emissionlow	0.963333	Positive	Prokaryota	Bacteroidota	UBA4372	sp	85.9	1.68	3772802	3.77	0.279271
94	Control_MidE.metabat.807	189	Emissionlow	0.926667	Positive	Prokaryota	Bacteroidota	Sodaliphilus	sp	88.26	4.84	2907118	2.91	0.224635
95	Control_MidE.vamb.1236	180	Emissionlow	0.938333	Positive	Prokaryota	Fibrobacterota	Fibrobacter	succinogenes_C	99.81	1.21	3639702	3.64	0.276667
96	Control_MidE.vamb.1335	65	Emissionlow	0.99	Positive	Prokaryota	Bacteroidota	Prevotella	sp	89.53	5.03	3109341	3.11	0.260573
97	Control_MidE.vamb.2656	149	Emissionlow	0.961667	Positive	Prokaryota	Bacteroidota	RF16	sp	87.12	1.79	2103845	2.1	0.170625
98	Control_MidE.vamb.2997	49	Emissionlow	0.991667	Positive	Prokaryota	Bacteroidota	Prevotella	sp	75.73	0.71	2567787	2.57	0.250781
99	Control_MidE.vamb.3575	64	Emissionlow	0.99	Positive	Prokaryota	Bacteroidota	Prevotella	sp	87.29	3.48	4075587	4.08	0.269167
100	Control_MidE.vamb.4233	183	Emissionlow	0.933333	Positive	Prokaryota	Bacteroidota	Prevotella	sp	73.5	7.08	2473489	2.47	0.229531
101	Control_MidE.vamb.4284	131	Emissionlow	0.97	Positive	Prokaryota	Bacteroidota	Prevotella	sp	92.12	0.35	3248786	3.25	0.280156
102	Control_MidE.vamb.4468	198	Emissionlow	0.923333	Positive	Prokaryota	Bacteroidota	UBA1711	sp	86.82	0.24	2405519	2.41	0.16974
103	Control_MidE.vamb.5834	126	Emissionlow	0.971667	Positive	Prokaryota	Bacteroidota	Cryptobacteroides	sp	82.11	6.41	2380058	2.38	0.211667
104	Control_MidE.vamb.9320	60	Emissionlow	0.991667	Positive	Prokaryota	Bacteroidota	RF16	sp	90.31	0.72	1937044	1.94	0.132552
105	HighE_A16_bin226	208	Emissionlow	0.911667	Positive	Prokaryota	Bacteroidota	Sodaliphilus	sp	95.13	2.95	3833874	3.83	0.227917
106	HighE_A60_bin265	88	Emissionlow	0.986667	Positive	Prokaryota	Bacteroidota	Cryptobacteroides	sp	85.42	2.44	1945608	1.95	0.149427
107	HighE_A60_bin614	47	Emissionlow	0.991667	Positive	Prokaryota	Bacteroidota	Prevotella	sp	90.41	6.95	4326248	4.33	0.290208
108	HighE_A63_bin207	167	Emissionlow	0.948333	Positive	Prokaryota	Bacteroidota	UBA4372	sp	70.96	7.66	2357637	2.36	0.150938
109	HighE_A67_bin151	43	Emissionlow	0.993333	Positive	Prokaryota	Bacteroidota	Cryptobacteroides	sp	70.89	1.06	1804977	1.8	0.17599
110	HighE_A67_bin467	220	Emissionlow	0.9	Positive	Prokaryota	Bacteroidota	F23-D06	sp	98.85	0.76	3691134	3.69	0.221719
111	HighE_A67_bin64	102	Emissionlow	0.981667	Positive	Prokaryota	Bacteroidota	Prevotella	sp	86.17	0.61	3106389	3.11	0.250052
112	LowE_A28_bin422	145	Emissionlow	0.963333	Positive	Prokaryota	Firmicutes	RUG626	sp	94.95	1.33	1737714	1.74	0.231563
113	LowE_A28_bin89	162	Emissionlow	0.95	Positive	Prokaryota	Bacteroidota	Prevotella	sp	94.79	3.24	4497063	4.5	0.298542
114	LowE_A28_bin97	172	Emissionlow	0.943333	Positive	Prokaryota	Firmicutes	Onthomonas	sp	83.42	1.12	1667223	1.67	0.20401
115	LowE_A43_bin272	56	Emissionlow	0.991667	Positive	Prokaryota	Bacteroidota	Cryptobacteroides	sp	84.99	2.4	2669414	2.67	0.224844
116	LowE_A59_bin190	32	Emissionlow	0.995	Positive	Prokaryota	Bacteroidota	Prevotella	sp	86.66	6.48	3474395	3.47	0.292031
117	LowE_A61_bin193	69	Emissionlow	0.988333	Positive	Prokaryota	Bacteroidota	Prevotella	sp	97.86	0.75	4035281	4.04	0.311302
118	LowE_A61_bin206	12	Emissionlow	0.998333	Positive	Prokaryota	Bacteroidota	Cryptobacteroides	sp	90.42	0.23	2126296	2.13	0.178594
119	LowE_A75_bin196	46	Emissionlow	0.993333	Positive	Prokaryota	Bacteroidota	Enterocola	sp	78.09	4.26	1978281	1.98	0.159635
120	LowE_A75_bin661	185	Emissionlow	0.933333	Positive	Prokaryota	Bacteroidota	UBA3663	sp	96.48	0.78	4826457	4.83	0.287344
121	Treatment_HighE.FMIC.metabat.129	54	Emissionlow	0.991667	Positive	Prokaryota	Actinobacteriota	UBA1367	sp	97.89	0.59	2575201	2.58	0.230833

122	Treatment_HighE.FMIC.metabat.38	92	Emissionlow	0.985	Positive	Prokaryota	Bacteroidota	RF16	sp	79.9	2.31	2081764	2.08	0.129531
123	Treatment_HighE.FMIC.metabat.422	119	Emissionlow	0.976667	Positive	Prokaryota	Actinobacteriota	RUG440	sp	75.2	6.88	2939682	2.94	0.187917
124	Treatment_HighE.FMIC.metabat.624	72	Emissionlow	0.988333	Positive	Prokaryota	Actinobacteriota	RUG440	sp	77.08	0.66	2724190	2.72	0.233802
125	Treatment_HighE.FMIC.metabat.700	29	Emissionlow	0.996667	Positive	Prokaryota	Bacteroidota	RF16	sp	74.03	6.57	2224992	2.22	0.155729
126	Treatment_HighE.FMIC.metabat.718	74	Emissionlow	0.988333	Positive	Prokaryota	Actinobacteriota	UBA1367	sp	94.67	1.24	2819132	2.82	0.232188
127	Treatment_HighE.FMIC.metabat.78	87	Emissionlow	0.986667	Positive	Prokaryota	Actinobacteriota	RUG440	sp	77.58	3.69	2506860	2.51	0.164531
128	Treatment_HighE.FMIC.vae_1015	93	Emissionlow	0.985	Positive	Prokaryota	Bacteroidota	UBA4334	sp	76.68	3.47	2685288	2.69	0.238594
129	Treatment_HighE.FMIC.vae_118	73	Emissionlow	0.988333	Positive	Prokaryota	Actinobacteriota	UBA1367	sp	79.09	4.11	2009272	2.01	0.151458
130	Treatment_HighE.FMIC.vae_1209	115	Emissionlow	0.978333	Positive	Prokaryota	Bacteroidota	UBA1711	sp	95.66	3.11	2399487	2.4	0.198125
131	Treatment_HighE.FMIC.vae_12217	105	Emissionlow	0.981667	Positive	Prokaryota	Bacteroidota	RF16	sp	74.39	2.94	1938674	1.94	0.140729
132	Treatment_HighE.FMIC.vae_128	188	Emissionlow	0.93	Positive	Prokaryota	Proteobacteria	Succinivibrio	sp	83.45	1.09	2901801	2.9	0.2325
133	Treatment_HighE.FMIC.vae_1557	128	Emissionlow	0.971667	Positive	Prokaryota	Bacteroidota	Prevotella	sp	79.99	4.03	2112751	2.11	0.204896
134	Treatment_HighE.FMIC.vae_17217	120	Emissionlow	0.976667	Positive	Prokaryota	Actinobacteriota	UBA1367	sp	90.37	3.84	2466413	2.47	0.235208
135	Treatment_HighE.FMIC.vae_1923	86	Emissionlow	0.986667	Positive	Prokaryota	Bacteroidota	Prevotella	sp	79.79	1.98	2833974	2.83	0.239427
136	Treatment_HighE.FMIC.vae_2102	124	Emissionlow	0.973333	Positive	Prokaryota	Actinobacteriota	RUG440	sp	84.44	3.83	3845006	3.85	0.189063
137	Treatment_HighE.FMIC.vae_2843	103	Emissionlow	0.981667	Positive	Prokaryota	Bacteroidota	Prevotella	sp	90.94	5.7	3887355	3.89	0.273281
138	Treatment_HighE.FMIC.vae_3145	52	Emissionlow	0.991667	Positive	Prokaryota	Bacteroidota	Prevotella	sp	82.84	3.93	3443462	3.44	0.267813
139	Treatment_HighE.FMIC.vae_32455	26	Emissionlow	0.996667	Positive	Prokaryota	Bacteroidota	UBA1711	sp	87.99	0.59	2470841	2.47	0.19901
140	Treatment_HighE.FMIC.vae_3778	68	Emissionlow	0.99	Positive	Prokaryota	Bacteroidota	UBA4372	sp	93.18	4.85	2504441	2.5	0.256719
141	Treatment_HighE.FMIC.vae_4731	130	Emissionlow	0.97	Positive	Prokaryota	Bacteroidota	Prevotella	sp	85.55	4.36	3531600	3.53	0.274271
142	Treatment_HighE.FMIC.vae_4762	206	Emissionlow	0.915	Positive	Prokaryota	Bacteroidota	Cryptobacteroides	sp	96.5	1.87	2230726	2.23	0.195208
143	Treatment_HighE.FMIC.vae_5146	28	Emissionlow	0.996667	Positive	Prokaryota	Bacteroidota	Limimorpha	sp	74.4	8.16	3484631	3.48	0.16099
144	Treatment_HighE.FMIC.vae_5436	195	Emissionlow	0.925	Positive	Prokaryota	Bacteroidota	UBA4372	sp	87.24	1.1	3071376	3.07	0.267083
145	Treatment_HighE.FMIC.vae_6706	57	Emissionlow	0.991667	Positive	Prokaryota	Bacteroidota	Cryptobacteroides	sp	94.73	0.38	2781699	2.78	0.230156
146	Treatment_HighE.FMIC.vae_6833	9	Emissionlow	0.998333	Positive	Prokaryota	Bacteroidota	Prevotella	sp	83	6.84	3797048	3.8	0.269688
147	Treatment_HighE.FMIC.vae_716	95	Emissionlow	0.983333	Positive	Prokaryota	Bacteroidota	Prevotella	sp	81.34	4.51	2463827	2.46	0.255729
148	Treatment_HighE.FMIC.vae_7946	20	Emissionlow	0.996667	Positive	Prokaryota	Bacteroidota	Prevotella	sp	83.55	5.3	2902180	2.9	0.264688
149	Treatment_HighE.FMIC.vae_885	137	Emissionlow	0.968333	Positive	Prokaryota	Bacteroidota	UBA4372	sp	85.17	2.87	4021021	4.02	0.290573
150	Treatment_HighE.FMIC.vae_8974	22	Emissionlow	0.996667	Positive	Prokaryota	Bacteroidota	Prevotella	sp	85.43	8.97	2775062	2.78	0.23474
151	Treatment_HighE.metabat.146	99	Emissionlow	0.983333	Positive	Prokaryota	Actinobacteriota	RUG440	sp	93.38	3.19	3236712	3.24	0.2225
152	Treatment_HighE.metabat.170	133	Emissionlow	0.97	Positive	Prokaryota	Bacteroidota	UBA4372	sp	84.77	4.28	3261800	3.26	0.278385
153	Treatment_HighE.metabat.30	66	Emissionlow	0.99	Positive	Prokaryota	Bacteroidota	Prevotella	sp	76.85	6.83	2744962	2.74	0.198802
154	Treatment_HighE.metabat.478	8	Emissionlow	1	Positive	Prokaryota	Bacteroidota	UBA4372	sp	74.03	7.19	3689618	3.69	0.237344
155	Treatment_HighE.metabat.626	116	Emissionlow	0.978333	Positive	Prokaryota	Bacteroidota	RF16	sp	70.73	9.51	2017806	2.02	0.107188
156	Treatment_HighE.metabat.661	214	Emissionlow	0.905	Positive	Prokaryota	Bacteroidota	Cryptobacteroides	sp	69.04	8.67	1813559	1.81	0.147708
157	Treatment_HighE.metabat.715	83	Emissionlow	0.986667	Positive	Prokaryota	Bacteroidota	Prevotella	sp	70.89	7.39	2410429	2.41	0.252708
158	Treatment_HighE.metabat.729	159	Emissionlow	0.951667	Positive	Prokaryota	Bacteroidota	Sodaliphilus	sp	93.53	4.74	3787617	3.79	0.221042
159	Treatment_HighE.metabat.763	217	Emissionlow	0.903333	Positive	Prokaryota	Actinobacteriota	RUG721	sp	96.25	2.93	2141705	2.14	0.236042
160	Treatment_HighE.metabat.772	177	Emissionlow	0.938333	Positive	Prokaryota	Bacteroidota	Sodaliphilus	sp	78.37	4.83	3401458	3.4	0.206875
161	Treatment_HighE.metabat.825	187	Emissionlow	0.931667	Positive	Prokaryota	Bacteroidota	Cryptobacteroides	sp	83.03	7.12	2862612	2.86	0.206615
162	Treatment_HighE.metabat.848	199	Emissionlow	0.923333	Positive	Prokaryota	Bacteroidota	UBA3663	sp	83.58	6.09	4338055	4.34	0.268906

163	Treatment_HighE.metabat.873	109	Emissionlow	0.98	Positive	Prokaryota	Bacteroidota	Prevotella	sp	70.95	6.19	3409990	3.41	0.226615
164	Treatment_HighE.vamb.1224	10	Emissionlow	0.998333	Positive	Prokaryota	Bacteroidota	Prevotella	sp	80.88	8.49	3879659	3.88	0.254219
165	Treatment_HighE.vamb.1671	108	Emissionlow	0.98	Positive	Prokaryota	Bacteroidota	Prevotella	sp	99.03	2.79	3392604	3.39	0.295156
166	Treatment_HighE.vamb.2080	21	Emissionlow	0.996667	Positive	Prokaryota	Bacteroidota	Prevotella	sp	88.19	7.1	3582772	3.58	0.273958
167	Treatment_HighE.vamb.2657	16	Emissionlow	0.998333	Positive	Prokaryota	Bacteroidota	Prevotella	sp	91.53	3.05	2445359	2.45	0.205
168	Treatment_HighE.vamb.37121	44	Emissionlow	0.993333	Positive	Prokaryota	Bacteroidota	Cryptobacteroides	sp	94.73	0.38	2781699	2.78	0.230156
169	Treatment_HighE.vamb.6249	61	Emissionlow	0.991667	Positive	Prokaryota	Bacteroidota	Prevotella	sp	95.52	1.14	2883898	2.88	0.277031
170	Treatment_HighE.vamb.6483	104	Emissionlow	0.981667	Positive	Prokaryota	Bacteroidota	F23-D06	sp	90.12	3.26	3134949	3.13	0.197917
171	Treatment_HighE.vamb.9748	78	Emissionlow	0.986667	Positive	Prokaryota	Bacteroidota	Prevotella	sp	75.02	7.33	2249236	2.25	0.19526
172	Treatment_LowE.FMIC.metabat.259	117	Emissionlow	0.976667	Positive	Prokaryota	Bacteroidota	Prevotella	sp	89.43	1.85	3106808	3.11	0.286302
173	Treatment_LowE.FMIC.metabat.6	173	Emissionlow	0.941667	Positive	Prokaryota	Bacteroidota	Prevotella	sp	79.42	0.41	2835653	2.84	0.26099
174	Treatment_LowE.FMIC.metabat.794	182	Emissionlow	0.935	Positive	Prokaryota	Firmicutes	GCA-900199385	sp	88.43	1.96	3152946	3.15	0.252813
175	Treatment_LowE.FMIC.metabat.902	80	Emissionlow	0.986667	Positive	Prokaryota	Bacteroidota	Prevotella	sp	86.95	9.15	2947759	2.95	0.256823
176	Treatment_LowE.FMIC.vae_10061	178	Emissionlow	0.938333	Positive	Prokaryota	Actinobacteriota	UBA9715	sp	81.33	1.83	2712128	2.71	0.220625
177	Treatment_LowE.FMIC.vae_1055	194	Emissionlow	0.925	Positive	Prokaryota	Bacteroidota	UBA4372	sp	74.45	2.49	2488040	2.49	0.211823
178	Treatment_LowE.FMIC.vae_10753	215	Emissionlow	0.905	Positive	Prokaryota	Bacteroidota	Limimorpha	sp	71.19	5.78	2416649	2.42	0.159063
179	Treatment_LowE.FMIC.vae_1426	144	Emissionlow	0.963333	Positive	Prokaryota	Bacteroidota	Prevotella	sp	83.69	4	3280286	3.28	0.255
180	Treatment_LowE.FMIC.vae_1492	106	Emissionlow	0.98	Positive	Prokaryota	Bacteroidota	Prevotella	sp	80.73	2.63	2471551	2.47	0.235052
181	Treatment_LowE.FMIC.vae_1546	45	Emissionlow	0.993333	Positive	Prokaryota	Bacteroidota	RF16	sp	73.59	2.51	1911863	1.91	0.138906
182	Treatment_LowE.FMIC.vae_18018	138	Emissionlow	0.966667	Positive	Prokaryota	Bacteroidota	Prevotella	sp	85.33	1.23	2472693	2.47	0.203021
183	Treatment_LowE.FMIC.vae_18068	58	Emissionlow	0.991667	Positive	Prokaryota	Bacteroidota	Cryptobacteroides	sp	88.87	0.43	2006090	2.01	0.184375
184	Treatment_LowE.FMIC.vae_18821	37	Emissionlow	0.995	Positive	Prokaryota	Bacteroidota	Prevotella	sp	81.53	0.48	2381819	2.38	0.271615
185	Treatment_LowE.FMIC.vae_236	196	Emissionlow	0.925	Positive	Prokaryota	Bacteroidota	UBA6382	sp	97.67	1.23	2842637	2.84	0.258958
186	Treatment_LowE.FMIC.vae_3394	70	Emissionlow	0.988333	Positive	Prokaryota	Bacteroidota	Prevotella	sp	95.59	5.68	3727923	3.73	0.276615
187	Treatment_LowE.FMIC.vae_370	41	Emissionlow	0.993333	Positive	Prokaryota	Bacteroidota	Prevotella	sp	72.12	3.11	3675317	3.68	0.249063
188	Treatment_LowE.FMIC.vae_3880	156	Emissionlow	0.953333	Positive	Prokaryota	Bacteroidota	Cryptobacteroides	sp	71.23	3.47	1944377	1.94	0.13776
189	Treatment_LowE.FMIC.vae_3946	96	Emissionlow	0.983333	Positive	Prokaryota	Bacteroidota	Prevotella	sp	81.44	4.08	3410588	3.41	0.246979
190	Treatment_LowE.FMIC.vae_5234	40	Emissionlow	0.995	Positive	Prokaryota	Bacteroidota	Prevotella	bryantii	99.56	2.25	3361169	3.36	0.313333
191	Treatment_LowE.FMIC.vae_6524	67	Emissionlow	0.99	Positive	Prokaryota	Verrucomicrobiota	RUG572	sp	77.8	8.96	4871871	4.87	0.279063
192	Treatment_LowE.FMIC.vae_6687	218	Emissionlow	0.903333	Positive	Prokaryota	Bacteroidota	Cryptobacteroides	sp	83.48	3.33	2279065	2.28	0.206979
193	Treatment_LowE.FMIC.vae_7547	3	Emissionlow	1	Positive	Prokaryota	Bacteroidota	Cryptobacteroides	sp	77.83	5.98	2260661	2.26	0.192031
194	Treatment_LowE.FMIC.vae_841	200	Emissionlow	0.921667	Positive	Prokaryota	Bacteroidota	Prevotella	sp	91.3	3.36	3087819	3.09	0.285052
195	Treatment_LowE.FMIC.vae_9548	207	Emissionlow	0.913333	Positive	Prokaryota	Bacteroidota	Prevotella	sp	84.06	3.54	3045036	3.05	0.270052
196	Treatment_LowE.metabat.224	107	Emissionlow	0.98	Positive	Prokaryota	Bacteroidota	Prevotella	sp	69.59	5.34	2072091	2.07	0.204427
197	Treatment_LowE.metabat.537	35	Emissionlow	0.995	Positive	Prokaryota	Bacteroidota	Prevotella	sp	85.95	1.82	3155974	3.16	0.276198
198	Treatment_LowE.metabat.625	219	Emissionlow	0.901667	Positive	Prokaryota	Bacteroidota	UBA4372	sp	69.88	4.48	2135455	2.14	0.191771
199	Treatment_LowE.metabat.645	171	Emissionlow	0.943333	Positive	Prokaryota	Firmicutes	Limivicinus	sp	98.76	0.3	3240912	3.24	0.257292
200	Treatment_LowE.metabat.65	148	Emissionlow	0.961667	Positive	Prokaryota	Bacteroidota	Cryptobacteroides	sp	78.69	1.68	2077965	2.08	0.189688
201	Treatment_LowE.metabat.881	210	Emissionlow	0.91	Positive	Prokaryota	Firmicutes	GCA-900199385	sp	90.31	2.34	3210056	3.21	0.283438
202	Treatment_LowE.vamb.1382	50	Emissionlow	0.991667	Positive	Prokaryota	Bacteroidota	Prevotella	sp	89.03	2.06	3455045	3.46	0.291354
203	Treatment_LowE.vamb.1606	153	Emissionlow	0.956667	Positive	Prokaryota	Bacteroidota	Prevotella	sp	97.9	0.12	3872237	3.87	0.327448

204	Treatment_LowE.vamb.2135	114	Emissionlow	0.978333	Positive	Prokaryota	Bacteroidota	Prevotella	sp	97.58	4.41	4482655	4.48	0.314271
205	Treatment_LowE.vamb.2338	81	Emissionlow	0.986667	Positive	Prokaryota	Bacteroidota	Prevotella	sp	86.02	1.82	2883754	2.88	0.275573
206	Treatment_LowE.vamb.3397	132	Emissionlow	0.97	Positive	Prokaryota	Bacteroidota	Cryptobacteroides	sp	73.06	2.86	1966237	1.97	0.159271
207	Treatment_LowE.vamb.4239	139	Emissionlow	0.966667	Positive	Prokaryota	Bacteroidota	Prevotella	sp	83.71	3.48	3466071	3.47	0.255156
208	Treatment_LowE.vamb.4899	85	Emissionlow	0.986667	Positive	Prokaryota	Bacteroidota	Prevotella	sp	84.38	2.86	3486295	3.49	0.256042
209	Treatment_LowE.vamb.5612	90	Emissionlow	0.985	Positive	Prokaryota	Bacteroidota	Cryptobacteroides	sp	78.95	7.71	2000479	2	0.170729
210	Treatment_LowE.vamb.6446	19	Emissionlow	0.996667	Positive	Prokaryota	Bacteroidota	Prevotella	sp	80.37	2.77	2163778	2.16	0.220885
211	Treatment_MidE.FMIC.metabat.456	209	Emissionlow	0.911667	Positive	Prokaryota	Bacteroidota	RF16	sp	99.24	1.48	2610268	2.61	0.184323
212	Treatment_MidE.FMIC.vae_4751	176	Emissionlow	0.941667	Positive	Prokaryota	Bacteroidota	UBA2918	sp	81.36	2.26	2688544	2.69	0.270052
213	Treatment_MidE.FMIC.vae_5433	174	Emissionlow	0.941667	Positive	Prokaryota	Bacteroidota	UBA1711	sp	90.81	0.39	2700670	2.7	0.185833
214	Treatment_MidE.FMIC.vae_5621	136	Emissionlow	0.968333	Positive	Prokaryota	Bacteroidota	RF16	sp	79.45	8.95	1851721	1.85	0.13
215	Treatment_MidE.metabat.460	143	Emissionlow	0.965	Positive	Prokaryota	Bacteroidota	Prevotella	sp	76.12	3.2	2434110	2.43	0.259896
216	Treatment_MidE.metabat.506	63	Emissionlow	0.99	Positive	Prokaryota	Bacteroidota	Prevotella	sp	77.98	6.81	2597243	2.6	0.215417
217	Treatment_MidE.metabat.515	129	Emissionlow	0.97	Positive	Prokaryota	Bacteroidota	Prevotella	sp	81.32	3.88	2776184	2.78	0.22875
218	Treatment_MidE.vamb.2602	112	Emissionlow	0.978333	Positive	Prokaryota	Bacteroidota	Prevotella	sp	62.97	6.34	2275413	2.28	0.203854
219	Treatment_MidE.vamb.5417	216	Emissionlow	0.903333	Positive	Prokaryota	Bacteroidota	Sodaliphilus	sp	73.56	0.73	3008852	3.01	0.186302
220	Treatment_MidE.vamb.701	152	Emissionlow	0.958333	Positive	Prokaryota	Bacteroidota	Prevotella	sp	84.29	6.54	3155906	3.16	0.262188

Table S5 Distribution of Samples Across Animal Breeds

Breed	LEC	HEC	Total (Samples)
Aberdeen Angus X	9	9	18
Luing	4	5	9
Total (Samples)	13	14	27

References

Kobel, C. M., Leu, A., Vera-Ponce de León, A., Øyås, O., Lai, W., Altshuler, I., Hagen, L. H., Wollenberg, R. D., Bakshani, C. R., Willats, W. G., Nicoll, L., McIlroy, S. J., Hvidsten, T. R., Schmidt, O., Greening, C., Tyson, G. W., Roehe, R., Aho, V. T. E., & Pope, P. B. (2024). Protozoal populations drive system-wide variation in the rumen microbiome. *bioRxiv*, 2024.2012. 2005.626740.
